# A Review of Biomimetic and Biodegradable Magnetic Scaffolds for Bone Tissue Engineering and Oncology

**DOI:** 10.3390/ijms24054312

**Published:** 2023-02-21

**Authors:** Gheorghe Paltanea, Veronica Manescu (Paltanea), Iulian Antoniac, Aurora Antoniac, Iosif Vasile Nemoianu, Alina Robu, Horatiu Dura

**Affiliations:** 1 Faculty of Electrical Engineering, University Politehnica of Bucharest, 313 Splaiul Independentei, District 6, 060042 Bucharest, Romania; 2 Faculty of Material Science and Engineering, University Politehnica of Bucharest, 313 Splaiul Independentei, District 6, 060042 Bucharest, Romania; 3 Academy of Romanian Scientists, 54 Splaiul Independentei, 050094 Bucharest, Romania; 4 Faculty of Medicine, Lucian Blaga University of Sibiu, 550169 Sibiu, Romania

**Keywords:** bone tissue engineering, magnetic scaffolds, magnetic nanoparticles, regenerative medicine, cancer therapy, magnetic hyperthermia, photothermal therapy

## Abstract

Bone defects characterized by limited regenerative properties are considered a priority in surgical practice, as they are associated with reduced quality of life and high costs. In bone tissue engineering, different types of scaffolds are used. These implants represent structures with well-established properties that play an important role as delivery vectors or cellular systems for cells, growth factors, bioactive molecules, chemical compounds, and drugs. The scaffold must provide a microenvironment with increased regenerative potential at the damage site. Magnetic nanoparticles are linked to an intrinsic magnetic field, and when they are incorporated into biomimetic scaffold structures, they can sustain osteoconduction, osteoinduction, and angiogenesis. Some studies have shown that combining ferromagnetic or superparamagnetic nanoparticles and external stimuli such as an electromagnetic field or laser light can enhance osteogenesis and angiogenesis and even lead to cancer cell death. These therapies are based on *in vitro* and *in vivo* studies and could be included in clinical trials for large bone defect regeneration and cancer treatments in the near future. We highlight the scaffolds’ main attributes and focus on natural and synthetic polymeric biomaterials combined with magnetic nanoparticles and their production methods. Then, we underline the structural and morphological aspects of the magnetic scaffolds and their mechanical, thermal, and magnetic properties. Great attention is devoted to the magnetic field effects on bone cells, biocompatibility, and osteogenic impact of the polymeric scaffolds reinforced with magnetic nanoparticles. We explain the biological processes activated due to magnetic particles’ presence and underline their possible toxic effects. We present some studies regarding animal tests and potential clinical applications of magnetic polymeric scaffolds.

## 1. Introduction

Bone tissue engineering (BTE) is an important topic in orthopedic [1] and craniofacial surgery [2,3]. To restore defects due to prosthetic implants and degenerative diseases such as osteoarthritis, osteoporosis, Paget’s disease, or osteogenesis imperfecta, biomimetic scaffolds can be involved [4,5,6,7,8]. Bone reconstruction is a complex process starting from inflammation, regeneration, and remodeling, each with its unique physical and biological mechanisms. Within this process, an important aspect is the action of the stem cells combined with growth factors or cytokines. Mesenchymal stem cells (MSCs) usually differentiate into osteoblasts, and hematopoietic stem cells (HSCs) are directly linked to osteoclast formation [9]. These two types of cells are essential for the formation and remodeling process of new bone. In the case of small bone defects, the healing phenomenon is spontaneous, but some supplementary interventions are required in the case of large defects [10,11,12]. Schemitsch [13] proposed a classification of bone defects. He considered that small defects are characterized by 50% cortical circumference loss and a defect size of less than 2 cm, intermediate defects consist of a cortical circumference loss higher than 50% and a defect size between 2 and 6 cm, and large defects exhibit a size greater than 6 cm [14]. Autografts or allografts are harvested using an invasive procedure associated with high risk of infection or disease transmission; even graft rejection can occur [15,16]. Usually, bone from the iliac crest, autologous vascularized fibular graft, or allograft is used. Another classical solution consists of the use of bone transport fixators; however, this method is linked to an increased healing time and pain. Autografts represent the gold standard in orthopedy, but this method is not always safe due to donor site morbidity effects and limited availability [17,18]. Regarding allografts, immunogenic responses or vascularization graft absence should be taken into account [19]. Grafting techniques are expensive, and due to high graft demand on the worldwide market, problems in bone defect treatments are foreseen [20,21,22,23].

### 1.1. Scaffolds for Bone Tissue Engineering

**Figure 1:**
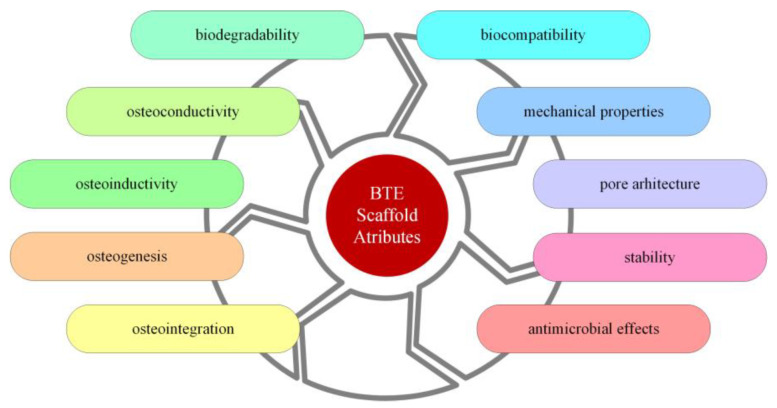
The most important attributes of a scaffold.

Bone scaffolds can be defined as artificial platforms dedicated to supporting and repairing a defect. A scaffold is necessary when an organ or a tissue is damaged, and in these cases, a three-dimensional (3D) structure is indicated. The most important properties and design features for a scaffold are biocompatibility, mechanical properties, biodegradability, pore size and interconnectivity, osteoinductivity, porosity, stability, antimicrobial effects, osteoconductivity, osteointegration, and osteogenesis, as depicted in Figure 1 [24,25,26,27].

For 3D scaffolds, a few criteria must be met before qualifying as an ideal implant. First, the scaffolds should have sufficient porosity to allow for tissue growth, adequate signaling, cellular ingress, and vascularization [28,29,30]. However, it is important to note that the mechanical properties of scaffolds are inversely proportional to their porosity. Therefore, recent studies [31,32] have recommended that scaffolds with a porosity of 200–350 μm are suitable for bone tissue regeneration. In the case of small bone defects, two-dimensional structures can be used as scaffolds to facilitate better interaction between cells and implant biomaterials [33].

After the scaffold is implanted, it is expected to provide a structure that is beneficial for cell proliferation, adhesion, and differentiation to create an adequate biomechanical medium for tissue regeneration, permit the dissemination of oxygen and nutritional substances, and to allow for the encapsulation of cells that will be released and combined with growth factors [34,35]. Scaffolds can be very useful for delivery of drugs and cells and, in the case of organ disease or failure, can sometimes be used to restore normal functionality. In bone tissue engineering, it is well known that bone-like porous structures ensure blood circulation, nutrient movement, and a combination of osteogenic cells and bioactive substances, which promote mineralization and angiogenesis in the transplanted graft. Chemical composition and topological aspects strongly influence scaffold surface properties, which are essential in cell adhesion and proliferation. The implant surface is the main boundary between biomaterial and tissue [36,37,38]. Surface roughness is considered a critical factor in osteoblasts’ adhesion and differentiation, and the mechanical properties of the scaffold must be similar to those of human bone to ensure successful and healthy bone grafting [39,40]. The implant must support the bone ingrowth process until the new bone can sustain itself. The efficacity of the regenerative process plays an important role through pore distribution, exposed surface area, the material’s porosity, the rate of cell penetration within the scaffold volume, and the extracellular matrix (ECM) architecture [41].

### 1.2. Biomaterials Used for Bone Tissue Engineering

Research has been focused on different types of scaffolds that show biological components [42,43]. These implants are, unfortunately, expensive, so scaffolds that do not contain so-called “biologics” provide significant advantages. They are adequately manufactured to collect and recruit cells from the tissue placed in the scaffold vicinity to enhance new bone formation. The use of adequate bioactive agents can sometimes be helpful for recruitment of cells with osteogenic characteristics [42,44]. As a result, the of mineralized matrices occurs in the entire implant structure [45,46]. A high amount improves the regenerative process. Angiogenesis is another essential aspect that sustains the needs of the new tissue [47].

Bioactive materials such as bioglass and calcium-phosphate-based ceramics are usually used in bone tissue engineering. They interact with natural tissue through an ion-exchange reaction, which leads to the formation of an active apatite layer on the scaffold [48,49]. Hydroxyapatite and tricalcium phosphate are biodegradable and begin to dissolve when introduced into human or animal bodies. Due to an increased similarity with human bone, scaffolds can be manufactured from bioactive ceramics that are corrosion-resistant, osteoconductive, and biocompatible [42]. Their main disadvantage is related to the fact that they are brittle and porous, and an increased risk of fracture can be foreseen. The most commonly used ceramics in BTE are hydroxyapatite, calcium phosphates, and β—tricalcium phosphate.

Another important class of materials is bioactive glasses. They have a composition based on SiO_2_, P_2_O_5_, and B_2_O_3_. The silanol groups result from SiO_2_ dissolution and precipitate into a silica layer that sustains the migration of phosphate and calcium ions, leading to the appearance of a layer of calcium phosphate [50,51]. In some bioactive glasses, partial replacement of SiO_2_ with B_2_O_3_ generates borosilicate or borate glasses that exhibit a controllable biodegradation rate. A faster degradation rate of the scaffold was noticed in the case of phosphate glasses that include Na_2_O and CaO. The advantages of bioactive glasses are controlled resorbability and osteoconduction. The main drawbacks are that the mechanical properties of the glass have values that differ from those of human bone and that the material must be tuned to control its degradation rate and ion release to avoid toxicity. Another disadvantage of bioactive glass was observed when 3D porous scaffolds were made; a crystallization phenomenon was identified during the sintering step [52]. As a result, the reduced compressive strength of the implant was put in evidence, which makes these types of materials suitable for scaffolds dedicated to use in low-load defect locations or as part of a composite structure with polymers or bioactive ceramics [53].

One of the most used materials in scaffold manufacturing is polymers, which can have natural or synthetic origins. The main polymeric implant properties are divided into three categories based on processing conditions, their intrinsic nature, and the final product. The intrinsic properties, such as density, solubility, crystallinity, transition temperature, mechanical properties, transparency, electromagnetic behavior, etc., depend on chemical composition and structure [54,55]. The viscosity, the melt strength, and melt flow index are considered the main processing characteristics and put in evidence the material behavior during the production process. The product’s properties combine those mentioned above and include esthetic properties, environmental behavior, and degradation conditions [56]. Polymeric scaffold biodegradability is very important and is defined as a gradual breakdown process of the material. There are two main biodegradable polymers: stepwise polycondensation and ring-opening polymerization materials. The first group includes polysaccharides and proteins [57], and the second contains aliphatic and aromatic polyesters. Most natural polymers are degraded by different enzymes. Polysaccharide-based biomaterials are degraded by amylases and lysosomes inside the human body. Many synthetic polymers are degraded by a hydrolytic process. The most common non-biological degradation processes are hydrolysis and erosion. The mechanical properties of polymers are influenced by molecular weight and crystallinity grade, which are directly linked to the degradation process of the material [58,59,60]. To obtain a successful treatment in the case of biodegradable polymers, it is important to maintain adequate mechanical strength to reconstruct load-bearing tissues such as bone. Rheological parameters such as Young’s modulus, flexural modulus, maximum strain, and tensile/compressive strength are always measured when a new implant enters the market [61]. The advantages of synthetic polymeric implants are that they can be manufactured under controlled conditions, and as a direct consequence, their degradation rate, mechanical properties, and porosity can be modified in accordance with different medical applications [62,63]. They can be produced in large quantities and can exhibit a homogenous structure. Better interaction with cells characterizes natural polymers, but they are found in limited quantity [64]. Their main drawback is that their properties cannot be controlled, as in the case of synthetic polymers, their toxicity must be carefully addressed [65].

Magnetic nanoparticles (MNPs) can be incorporated into scaffolds manipulated in situ under electromagnetic forces [66,67,68]. Due to the influence of magnetic field, these implants offer the possibility of increased osteogenesis and angiogenesis at large bone defect sites [69]. Many literature studies have proven that scaffolds reinforced with MNPs support the differentiation and proliferation of osteoblasts in the presence and absence of a magnetic field by activating dedicated signal pathways [44,70,71,72,73,74,75]. Treatment of bone tumors with methods such as magnetic hyperthermia or photothermal therapy is also possible.

**Figure 2:**
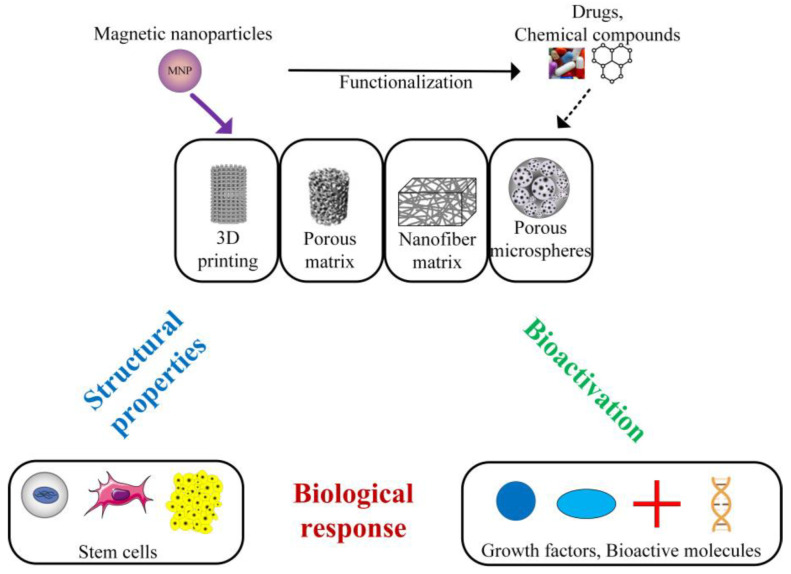
Main components of a magnetic scaffold used in BTE.

This review focuses on biodegradable magnetic polymeric scaffolds by providing insight into the biomaterials used in implant manufacturing; mechanical, thermal, and magnetic properties of the scaffolds; the influence of magnetic field on cells; biocompatibility; and osteogenic effects. Furthermore, we discuss issues related to the toxicity of magnetic nanoparticles, *in vitro* and *in vivo* analysis, and potential clinical applications of magnetic scaffolds. The main magnetic scaffold components are presented in Figure 2 [76,77], taking into account the scaffold geometry and shape and its combination with stem cells [78,79], growth factors or bioactive molecules [80], chemical compounds, and drugs [81].

## 2. Biomaterials Used in Magnetic Polymeric Scaffolds Designed for Bone Regeneration

**Figure 3:**
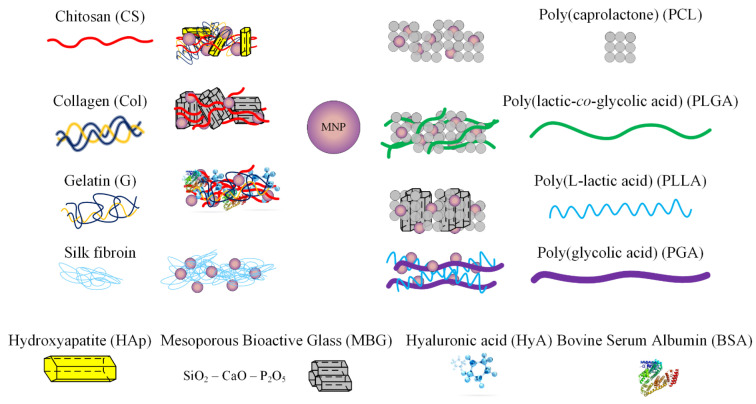
Types of biomaterials and scaffolds used in BTE.

Biomaterials for scaffolds must possess the ability to present biomimicry by taking into account the properties mentioned above, as explained in Section 1.2. Many studies have been conducted to determine the best material combinations to obtain an enhanced osteogenic and angiogenic effect of the scaffold when implanted in the human body [2,42,69,82,83]. In this direction, magnetic nanoparticles can be successfully combined with polymer scaffolds, obtaining an increased osteogenic effect on the stem cells [44,70,71,72,73,75]. Through MNPs, drugs or bioactive agents can be directly guided to the defect site to help in bone regeneration [84]. Figure 3 shows several types of biomaterials and scaffolds used in bone tissue engineering.

Table 1 presents studies conducted on magnetic scaffolds based on different biopolymers and magnetic nanoparticles [85,86,87,88,89,90,91,92,93,94,95,96,97].

### 2.1. Magnetic Scaffold Manufacturing Technique

The scaffold manufacturing technique is chosen according to the following criteria. The chemical properties of the material must not be modified during the production process to negatively influence the implant’s clinical use or alter its biocompatibility [98]. There are two types of manufacturing technologies: conventional and the advanced techniques. Conventional techniques are based on subtractive routes that consist of material removal from an initial bulk volume to obtain the desired shape of the implant. The main drawback is that a random architecture of the scaffolds results [98]. These technologies imply the use of organic solvents, which may harm cell functions and viability [99].

On the other hand, advanced methods permit the control of scaffold geometry and pore size. Tunable mechanical properties characterize the implants, in accordance with the surrounding tissue attributes. These methods allow for compositional variation of different materials across the interface, surface, or volume of the scaffolds. In addition, they do not use toxic organic solvents, which is directly linked to increased scaffold biocompatibility [100].

Some of the most important techniques for the manufacture of polymeric scaffolds using conventional technologies are freeze drying, electrospinning, gas foaming, solvent-casting particulate leaching, and thermally induced phase separation.

Freeze drying is based on polymeric slurry production. After that, it is poured into a mold and frozen. The resulting ice crystals generate the scaffold pores, and lyophilization occurs once the slurry undergoes solidification. Scaffolds manufactured through freeze-drying exhibit a porous structure with low stiffness and small pores. The main disadvantages of this method are high energy consumption, the use of cytotoxic solvents, and the long duration of the procedure [100,101,102]. The electrospinning technique consists of an electric charge liquid jet used to generate, with the help of a syringe pump, fine polymeric fibers, creating a collector on a nanofibrous architecture. The system’s main components are a high-voltage power supply, a syringe pump, a spinner with a metallic needle, and a collector connected directly to the ground. The electric field strength overcomes the surface tension of the material droplet, and a charged liquid jet, which is continuously deformed by the electrostatic repulsion phenomenon, is deposited on the collector. Fibrous polymeric scaffolds are manufactured using this technology [103]. A drawback of this method is that it is linked to organic solvent use. Sponge-like scaffolds based on inert gases that pressurize molded polymers with fluoroform and water are obtained using gas foaming. The material becomes saturated and is characterized by gas bubbles. An advantage of this technique is the avoidance of toxic solvents, and the disadvantages are the heat developed during the compression molding process, isolated pores, and a continuous skin layer [104]. Solvent-casting particulate leaching requires a solvent containing a dissolved polymer solution to be mixed with specific diameter-sized salt particles. By evaporating the solvent, an embedded salt matrix is obtained. Using water, the salt leaches out, generating a highly porous structure. The advantages of this method are high porosity and a controllable pore diameter through salt particle size. The main drawbacks include residual solvent presence and scaffolds with a simple geometry [105,106]. In the case of the thermally induced phase separation method, the polymer solution is subjected to a low temperature, so a liquid–liquid phase separation is obtained. Two phases result: a polymer-rich phase and a polymer-poor phase. The polymer-poor phase is eliminated during solidification. A highly porous nanoscale structure is obtained [107].

The advanced methods are part of the class of rapid prototyping technologies that include selective laser sintering (SLS), selective laser melting (SLM), stereolithography (SL), fused deposition modeling (FDM), and binder jetting (BJ).

SLM and SLS are derived from the powder bed fusion class and can be used to obtain scaffolds with desired shape architecture and controlled porosity; however, small details such as sharp corners or complicated boundaries cannot be designed [108]. Through SLS, powder particles are bonded in thin layers under a high-power laser effect. The last formed layer is bounded to the previous layer as indicated in a predefined computer-aided design (CAD) file. The main drawbacks of this technology are the high operating temperature and the fact that residual powder must be removed [109,110,111]. SL includes a tank with a photosensitive liquid polymer placed in a thin layer on a movable built platform. The desired geometry layer is defined using an ultraviolet (UV) laser, the platform is lowered, and the process is repeated. This method is fast and provides a high resolution. Its drawbacks are brittleness and low mechanical strength of the scaffold [108]. FDM implies a molten thermoplastic material extruded through a nozzle to form a continuous thin filament printed on an imposed CAD pathway in a layer-by-layer procedure. Through this technique, a controlled porosity can be obtained [112]. The method does not require toxic solvents [113,114,115]. BJ technology is based on a deposited powder bed on which, using a printing head, a liquid binder solution that describes the required geometry is placed. The advantages of this method are the manufacture of scaffolds adapted to the patient’s anatomy or multilayered implants used for hybrid tissue regeneration. The unbounded powder removal, the limited pore size configuration, and the possibility of the binder being dissolved are the main drawbacks [2]. Three-dimensional bioprinting technology offers the possibility of including cells and differentiation or growth factors in the scaffold geometry. Its main drawback is that during the post-fabrication stages, the solvent must be entirely removed [116,117].

**Figure 4:**
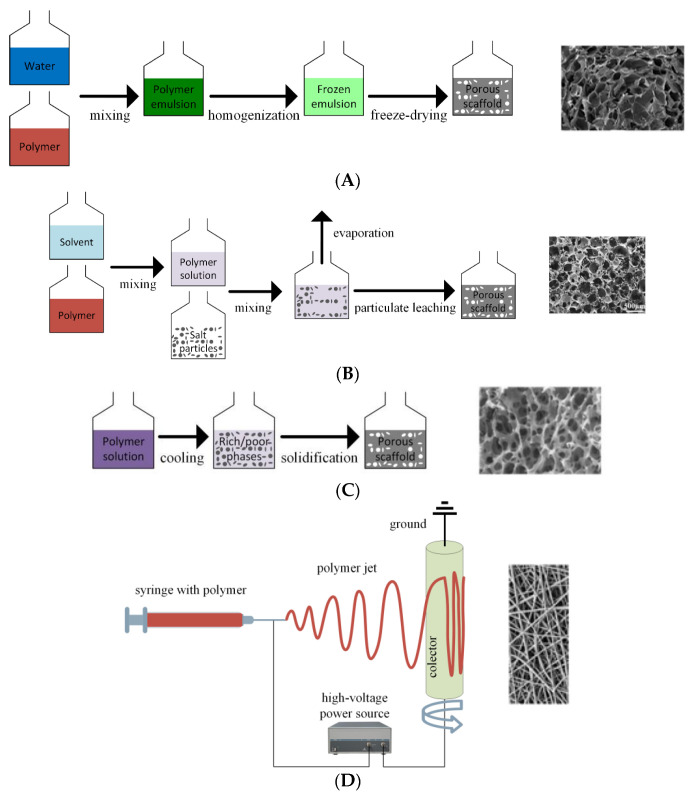

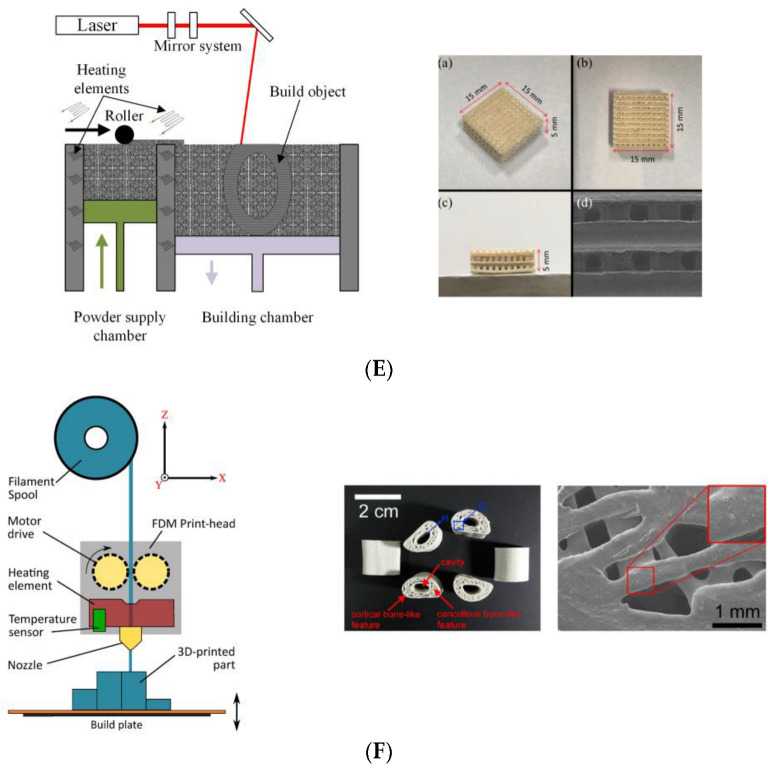
Examples of conventional and advanced preparation methods for polymeric scaffolds: (**A**) freeze drying [102]; (**B**) solvent-casting particulate leaching [106]; (**C**) thermally induced phase separation [102]; (**D**) electrospinning [102]; (**E**) selective laser sintering ((**a**–**c**) optical images and (**d**) SEM image) [2,111]; (**F**) fused deposition modeling [112,115] ((**A**–**D**) (**right**), (**E**), and (**F**) (**left**) are licensed under CC-BY 4.0; (**F**) (**right**) is adapted with permission from Xu, N. et al.; 3D artificial bones for bone repair prepared by computed-tomography-guided fused deposition modeling for bone repair; copyright 2023 American Chemical Society).

Figure 4 shows some of the conventional and advanced preparation methods and examples of obtained polymeric scaffolds.

Table 2 summarizes the advantages and disadvantages of scaffold manufacturing techniques.

As previously mentioned, MNPs can be inserted into polymeric scaffolds to enhance cell adhesion and differentiation or to apply regenerative or oncological treatments. They are fabricated using solvothermal, hydrothermal, coprecipitation, sol–gel, electrochemical, and laser pyrolysis methods [77]. Other preparation technologies reported in the literature are powder metallurgy, evaporation synthesis, laser ablation, and microbial methods [118]. The most used technology is precipitation from a solution. Magnetite (Fe_3_O_4_) is prepared based on an aqueous solution of Fe^3+^ and Fe^2+^ chloride combined with a base. Coprecipitation consists of a ferric and ferrous hydroxide suspension that is oxidized through different chemical substances (i.e., Fe^2+^ salt, nitrate ions, and a base) or a mixture of stoichiometric ferric and ferrous hydroxides that are aged in aqueous media. Other important methods are based on microemulsions, which generate nanoparticles with tunable sizes and distributions, reverse micelle solutions, or polyols, as explained in detail in [77].

Different methods have been reported in the literature to obtain magnetic scaffolds. Lu et al. [86] added SrFe_12_O_19_ nanoparticles prepared by the molten salt method and MBG microspheres in CS solution. After a stirring procedure, the samples were frozen under an external magnetic field, and finally, a freeze-drying step was applied. Cojocaru et al. [87] made magnetic scaffolds from natural biopolymers combined with Fe_3_O_4_ MNPs using a biomimetic coprecipitation method. Before this process, different concentrations of MNPs were added to the polymer solution, and after that, freeze-drying technology finished the production method. Samal et al. [89] designed silk scaffolds based on a salt-leaching procedure in which they infused MNPs under a static magnetic field effect generated by a permanent magnet. Dankova et al. [91] made a mixture of polycaprolactone, adding MNPs based on the dispersion method. Then, fibrous scaffolds were generated through the electrospinning method. De Santis et al. [92] designed, through rapid prototyping, 3D fully biodegradable magnetic scaffolds made from polycaprolactone reinforced with iron-doped hydroxyapatite (FeHAp) nanoparticles processed through a 3D fiber deposition method. They investigated the influence of FeHAp nanoparticle concentration to adapt the implants to dedicated medical applications such as advanced bone tissue engineering or magnetic hyperthermia.

The choice of the scaffold production method must consider the geometry of the required implant (because it should be patient-oriented), the toxicity grade of the MNPs, and its cost. Magnetic scaffolds represent an innovative approach for large bone treatments, and they are applicable for drug delivery, cell guidance based on magnetic force action, and cancer therapy.

### 2.2. Magnetic Nanoparticles Used for Scaffold Loading

**Figure 5:**
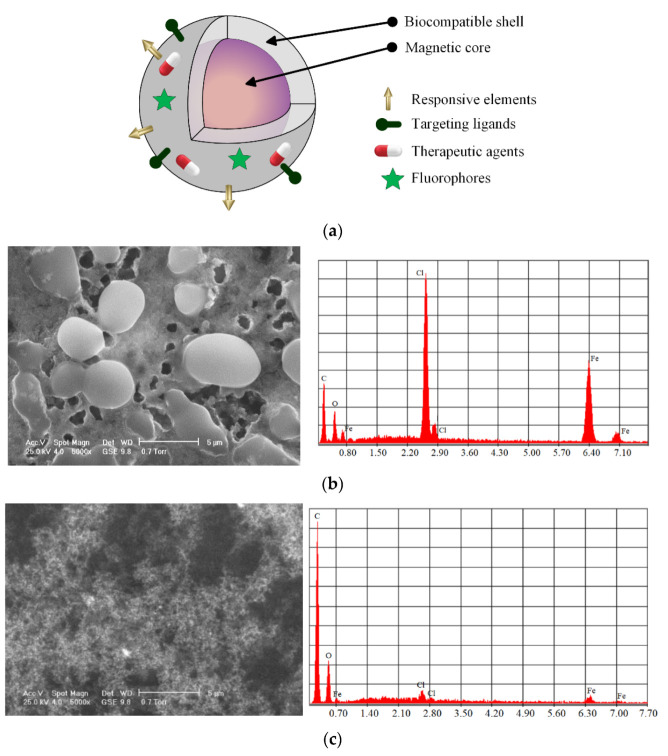
Representation of an MNP, SEM images, and elemental analysis of Fe_3_O_4_ nanoparticles prepared through coprecipitation method: (**a**) classic illustration of MNP structure; (**b**) SEM images and elemental analyses of uncoated MNPs [77]; (**c**) SEM images and elemental analyses of MNPs functionalized with chitosan [77] ((**b**,**c**) are licensed under CC-BY 4.0).

Magnetic nanoparticles exhibit unique material properties because they can be manipulated using an externally applied magnetic field. These nanomaterials are made from a magnetic core, which contains a different oxide of ferromagnetic metals such as iron (Fe), cobalt (Co), or nickel (Ni) that can be coated with a biocompatible material with unique properties for medical applications [77,119,120]. Figure 5a shows a classic scheme of a coated MNP that can be used for ligand transport, responsive elements, and fluorophores. Figure 5b,c show scanning electron microscopy (SEM) images and elemental analyses of Fe_3_O_4_ uncoated MNPs functionalized with chitosan.

The most common applications of MNPs are magnetic hyperthermia, magnetic drug targeting therapy, and as magnetic resonance imaging (MRI) contrast agents. The size of the magnetic particle represents the main parameter that separates ferromagnetic and superparamagnetic behavior. Below a critical diameter, the MNPs present a so-called superparamagnetic state characterized by a magnetic single-domain configuration with a sigmoidally shaped magnetization curve. High magnetic susceptibility and saturation magnetization values characterize superparamagnetic iron oxide nanoparticles (SPIONs). These nanoparticles are special MNPs extensively used in biomedicine [121,122]. They are biocompatible and chemically stable and exhibit environmentally friendly behavior. Superparamagnetic behavior is directly linked to the magnetic anisotropy of the MNPs measured along the easy magnetization axis of the particle, which is a direction characterized by a minimal value of magnetic anisotropy energy. In the case of spherical magnetic nanoparticles, the total magnetic anisotropy can be considered a barrier in the magnetization direction change [123]. At very low values of the particle diameter, the anisotropy energy is almost equal to the heat activation energy [124], and when the latter is increased, there is no preferential direction for the magnetic moment orientation. The behavior of SPIONs could be assimilated to that of paramagnetic atoms [125,126]. The value of the temperature at which the thermal activation is higher than the magnetic anisotropy energy is denoted in the literature as the blocking temperature [127,128].

It was previously shown that particles with a large diameter are more toxic than smaller particles when an alternating low-frequency magnetic field is applied [129]. At a diameter lower than 200 nm, the nanoparticles are not trapped in the sanguine system and are expelled through the mononuclear phagocyte system and hepatic filtration function [130,131].

The material surface must be modified to improve the drawbacks of magnetic nanoparticles, such as poor biodegradability, chemical instability, and moderate biocompatibility. One of the most used methods is MNP functionalization with different materials. Biofunctional molecules such as ligands, antibodies, or receptors can link different nanostructures of the human body to the magnetic core, making some treatments more efficient [132]. Another technology consists of the integration of SPIONs or, in general, MNPs with other metallic nanoparticles, which leads to so-called heterodimer structures. These unique materials permit the attachment of functional molecules to a specific surface part of the heterodimer that can bind to different receptors or act as agents in imaging techniques [133]. A direct application is a platform for bacterial detection [134,135].

The chemical stability and solubility of MNPs must be carefully controlled in biological media. It is well known that by incorporating MNPs into biodegradable polymeric scaffolds, owing to their hydrophilic nature, the implant wettability is improved, and increased cell adhesion and proliferation are observed [93]. The MNP concentration also plays an important role in the improvement of mechanical properties. Some studies have evidenced well-established MNP concentrations beyond which a decrease in mechanical strength is reported [136,137]. However, as an overall finding, the addition of MNPs can be linked to a reduction in the porosity percentage of PCL scaffolds and an increase in the porosity grade of chitosan or collagen implants [85,138]. When SPIONs are incorporated into polymeric scaffolds, because each particle is a single magnetic domain, the implant exerts a magnetic influence on the receptors placed on the cell membrane, activating the intracellular signaling pathways [88,137]. Due to the presence of magnetic induction, the cell cycle is accelerated, and osteogenic differentiation is put in evidence [91].

Modern magnetic scaffolds consist of a matrix made from different materials and magnetic nanoparticles chemically doped or physically loaded into the implant structure. The matrix is usually made from bioceramics, polymers, or hydrogels, and it is a suitable tool in regenerative medicine and anticancer therapy because the magnetic hyperthermia effect can be combined with the osteoinductive properties of the MNPs [139].

Table 3 shows the magnetization values of some MNPs incorporated in polymeric or composite matrices of different types of scaffolds used in bone tissue engineering.

### 2.3. Biopolymers for Magnetic Scaffolds

Biodegradable polymers can be used to manufacture the scaffold matrix in BTE. In the case of medical applications, increased attention is devoted to the cellular environment and the interaction between materials and cells [141,142]. Due to their specific properties, such as biodegradability, high porosity, important surface-to-volume ratio, and favorable mechanical properties, polymeric scaffolds have become among the most used implants for BTE [143,144].

#### 2.3.1. Natural Biopolymers

As a function of structure and monomeric units, there are three important polymer types: polysaccharide-based (e.g., chitosan, chitin, hyaluronic acid, and alginate), polypeptide- and protein-based (e.g., collagen, silk, and gelatin), and polynucleotide-based [25] polymers.

Members of the polysaccharide-based group are made from disaccharide or monosaccharide chains. Chitin and chitosan are characterized by non-toxicity, biocompatibility, and biodegradability properties [145]. They owe reactive species as hydroxy and amino groups, high charge density and exhibit broad hydrogen-bonding capabilities and a single chemical structure. Due to their reactive species, chitosan and chitin can be easily linked to different biomolecules to increase the biocompatibility of scaffolds. The biodegradation rate of these biopolymers depends on the acetyl content, and their *in vivo* breakdown occurs as a result of lysozymes. If chitosan is modified in an appropriate manner, scaffolds for bone regeneration can be produced [146]. Zhao et al. [85] prepared magnetic bioinspired micro/nanostructured composite scaffolds based on a chitosan/collagen organic matrix. They incorporated nanohydroxyapatite (nHAp) and Fe_3_O_4_ nanoparticles into scaffolds. The matrices were prepared by in situ crystallization and freeze-drying technique. *In vitro* analyses including physicochemical and biocompatibility tests proved that [CS/Col]/[Fe_3_O_4_/nHAp] magnetic implants were characterized by good structural and mechanical properties and were beneficial for cell adhesion and proliferation. Enhanced osteogenic differentiation due to the presence of MNPs was also noticed. Mineralization tests showed that the magnetic scaffolds have a very good in situ biomimetic mineralization process. Lu et al. [86] fabricated magnetic nanoparticles of SrFe_12_O_19_ that were incorporated in modified MBG/CS porous scaffolds. These implants proved to have beneficial properties against tumors with excellent bone regeneration effects. SrFe_12_O_19_ nanoparticles had an improved photothermal conversion property. Cojocaru et al. [87,117] made biopolymer–calcium phosphate composites with the inclusion of MNPs. As biopolymers, they used chitosan, hyaluronic acid, bovine serum albumin, and gelatin, and as MNPs, they used magnetite nanoparticles prepared by the coprecipitation method. The morphology of the magnetic scaffolds was investigated using scanning electron microscopy (SEM), Fourier transform infrared spectroscopy (FTIR), X-ray diffraction, and energy-dispersive X-ray spectroscopy. *In vitro* degradation analysis showed evidence of a slow degradation rate, and a biocompatibility test revealed no adverse effects on osteoblast cells. The mechanical properties of the scaffold were improved by increasing the MNP content.

Hyaluronic acid is a linear polysaccharide that can be found in many parts of the extracellular tissue [147]. It can be used in hydrogel or solution form to repair different body sites because HyA is a part of the connective tissue with an essential role in lubrification, cell differentiation, and growth. Hydrogels from HyA can be easily produced due to functional groups such as alcohols or carboxylic acids. It is well known that innovative scaffolds can be made from HyA, and they exhibit biodegradable and bioactive properties, showing non-specific protein adsorption. These scaffolds are very effective in tissue repair and growth via cell receptors [148]. Zheng et al. [149] provided a comprehensive review of hyaluronic-acid-based materials used in bone regeneration. Composite hydrogel systems have proven their efficiency due to good mechanical properties, high biocompatibility, and biodegradability. They can also be combined with MNPs for drug delivery and an enhanced osteogenesis process. HyA stimulates extracellular matrix microenvironments, promotes cellular activities, and can realize crosslinking action with other polymers, and MNPs help deliver drugs or growth factors. Three-dimensionally printed HyA scaffolds have proven to have a strong influence on the bone formation process.

Proteins and peptides are derived from α—L amino acids. The main drawbacks of polypeptide- and protein-based materials consist of their lack of processability and immunogenicity, but good biological properties and low mechanical strength characterize these biopolymers. Collagen scaffolds are already implemented in clinical treatments, and some are in trials [150]. Collagen degradation occurs as a result of two enzymes, i.e., collagenases and metalloproteinases, which produce an amino acid [151]. Mechanical properties of type I collagen must be tuned. Its limited chondrogenic capacity and significant shrinking must be improved for use in scaffold manufacturing. Usually, collagen is combined with HyA, CS, or chondroitin sulfate [152]. Collagen scaffolds are not used for load-bearing areas of the human body. Bianchi et al. [153] investigated the nanomechanical properties of newly formed bone four weeks after implantation surgery. This was performed through magnetic scaffold insertion into the trabecular bone of rabbit femoral condyles. The developed magnetic scaffolds contain NdFeB magnets combined with HAp/Col, with MNPs directly nucleated on the collagen fibers in the manufacturing process or introduced later. It was concluded that the second production technique led to better results because the mechanical properties of the neo-bone had similar values to those of native bone. The addition of MNPs to the final product has an important influence on the osteogenesis process.

Through disintegration or denaturation, insoluble collagen results in a gelatin degradation compound. Gelatin has poor mechanical properties and a high biodegradability rate, and due to active chemical groups such as amino and carboxyl acids, the degradation time can be increased through different chemical treatments [154,155]. Dashnyam et al. [88] prepared a novel magnetic scaffold based on gelatin–siloxane for bone tissue engineering by incorporating magnetite magnetic particles using the sol–gel process. Porous scaffolds were manufactured based on the freeze-drying method. With the addition of MNPs, the mechanical properties were highly improved, the scaffolds exhibited superparamagnetic behavior, and the saturation magnetization increased directly proportionally to the MNP content. The developed implants showed good bone bioactivity. The rapid growth of the apatite minerals crystals and osteogenic differentiation were put in evidence. Cellular mineralization increased when MNPs were added.

Silk is a natural protein-based biopolymer characterized by good mechanical properties, a controllable degradation rate, and high biocompatibility. Silk fibers have high strength, good durability, low weight, and high elasticity. Silk is made from two proteins. The first is called fibroin, which consists of a fibrous portion; the second is named sericin, which is soluble in water and contains 18 amino acids [156,157]. Samal et al. [89] developed biomimetic magnetic silk fibroin protein scaffolds. These implants were intended to be used in magnetic-field-assisted BTE. MNPs were introduced into scaffolds through the dip-coating technique. Good magnetic hyperthermia, improved osteogenic effects, cell adhesion, and proliferation were reported.

#### 2.3.2. Synthetic Biopolymers

Synthetic biopolymers are characterized by tunable properties and well-established structures and can be produced in different forms. They are much more easily manufactured than natural polymers but have a main drawback, i.e., bioinertia [158]. Many modern synthetic materials have mechanical and physiochemical properties similar to those of human bone. The main classes of synthetic biopolymers include poly(α—hydroxy esters) (poly(ε—caprolactone) (PCL), polylactic acid (PLA), polyglycolic acid (PGA), poly(L-lactide-co-glycolide) (PLGA)) and poly(ethers) (poly(ethylene oxide) (PEO), polyvinyl alcohol (PVA), poly(ethylene glycol) (PEG), and polyurethane (PU)). These are the most used materials, and they have an imposed Young’s modulus, degradation rate, and mechanical strength. The abovementioned synthetic biopolymers exhibit different levels of biocompatibility, biodegradability, and mechanical properties, and no single material envisages all the ideal properties for scaffold manufacturing [159].

Poly(ε—caprolactone) (PCL) is a biocompatible aliphatic and semicrystalline polymer that is very tough and hydrophobic [160]. The initial degradation process consists of non-enzymatic bulk hydrolysis of ester connections that are catalyzed with the help of carboxylic acid end groups. PCL can induce foreign body responses, which are evidenced by the giant cells and the presence of macrophages. To increase its biocompatibility, solutions such as surface functionalization or a blended formulation must be considered. The rate of deterioration is relatively slow and can be longer than two years [161]. Ganesh et al. [90] incorporated a multimodal contrast agent with HAp nanocrystals inside a poly(caprolactone) nanofibrous scaffold produced through electrospinning. Magnetic resonance was used to analyze the scaffold’s influence on tissue regeneration. The implant biocompatibility was put into evidence through *in vitro* tests with the help of human MSCs. Incorporating multifunctional hydroxyapatite nanoparticles (MF-nHAp) within the PCL nanofibers leads to the increased strength of the scaffold, good protein adsorption, proliferation, and differentiation of the cells. Dankova et al. [91] developed a nanofibrous scaffold through electrospinning from PCL and MNPs. The biocompatibility of the scaffold was put into evidence by taking into account the biomaterial influence on fibroblasts and MSCs. When the MNP percentage increases, a more critical stimulation of cell adhesion, proliferation, and differentiation were noticed. A gradual rise in the saturation magnetization was observed, and it was concluded that up to 10% wt. MNPs, the effects were contained in the biological range. De Santis et al. [92] used rapid prototyping (RP) technology to obtain 3D magnetic nanocomposite scaffolds made from a PCL matrix reinforced with iron-doped hydroxyapatite. It was noticed that by adding magnetic properties to biopolymers, an enhanced osteointegration process is obtained. Kim et al. [93] studied a classical magnetic scaffold made of PCL and MNPs. MNPs were produced by a surfactant mediation process and distributed in the PCL matrix. Superparamagnetic behavior was observed, and it was concluded that the incorporation of MNPs leads to high hydrophilicity and water swelling of scaffolds. Using acellular apatite-forming ability tests, a high mineral induction potential of the implant was revealed. The mechanical stiffness increased directly proportionally to the MNP content, and high cell adhesion and proliferation were observed during *in vitro* tests.

Unfortunately, PCL exhibits hydrophobic behavior, leading to reduced cell affinity and a low rate of tissue regeneration. To address this critical limitation, PCL can be combined with different polymers such as PLA or PLGA, and cell proliferation and adhesion can be improved [162]. The synthetic polymer PLGA was approved by the Food and Drug Administration (FDA) for clinical use. Scaffold structures made from this material have been developed, which have proven to be efficient if they have an adequate porosity grade characterized by precise contour geometrical dimensions and internal morphologies, which sustain cellular attachment and structure colonization [96]. In this case, the scaffold surface can be functionalized with bioactive substances or chemicals, and plasma treatment can be applied to increase implant efficacity. Chen et al. [94] optimized the interaction between seed cells and scaffold to ensure beneficial conditions for cell growth under natural biomimetic conditions. They have reported the manufacture of a magnetic [PLGA/PCL] scaffold made using electrospinning technology and layer-by-layer assembly of superparamagnetic iron oxide nanoparticles. These composite scaffolds exhibited increased hydrophilicity and a high value of elastic modulus. They have a good influence on the osteogenesis process of stem cells. It was concluded that the magnetic properties of implants are a key factor in enhancing osteogenic differentiation, which is important as a bioactive interface between cells and scaffolds. The results were compared with those obtained in the case of gold nanoparticles, and the authors concluded that using MNPs in scaffold production leads to an increased osteogenic effect and a high application potential in BTE. Zhang et al. [95] developed 3D MNPs combined with mesoporous bioactive glass/polycaprolactone ([MBG/PCL]/[Fe_3_O_4_]) composite scaffolds. *In vitro* bioactivity, chemotherapeutic drug delivery, mechanical strength, and magnetic heating effect were put in evidence. The produced scaffolds had uniform macropores of 400 μm, a high porosity grade of 60%, and good compressive strength of about 14 MPa. The incorporation of MNPs did not disturb the apatite mineralization process but provided the scaffolds with a high magnetic heating ability and enhanced osteogenesis-related gene expression. The authors concluded that these medical devices are essential in cancer therapy, and they can also stimulate new bone formation and angiogenesis.

PLA is a semicrystalline polymer with high biocompatibility, hydrophobic properties, biodegradability, and easy processability [163]. The degradation products that result are carbon dioxide and water, which are not harmful to the human body [164]. This polymer can be used in clinical practice as poly(L-lactic acid) (PLLA), poly(D,L-lactic acid) (PDLLA), and poly(D,L-lactide) (PDLA). Shuai et al. [96]. elaborated a PLLA/PGA scaffold made using the laser sintering method incorporated with Fe_3_O_4_ magnetic nanoparticles. A rigid enhancement effect of MNPs was put in evidence through an increase in compressive strength and modulus of about 70%. After *in vitro* and *in vivo* tests, the obtained results indicated enhanced angiogenesis and osteogenesis effects, fibrous tissue formation, and new bone development.

PGA is a linear aliphatic polyester not soluble for organic solvents because it has a high degree of crystallinity. PGA can break into glycolic acids, which can be combined with the tricarboxylic acid cycle, and expel products such as water and carbon dioxide [165]. PLGA is a well-known ring-opening copolymer of PGA and PLA that is biodegradable, has a low toxicity level, good mechanical properties, a controllable degradation rate, and favored cell adhesion and multiplication. In the BTE domain, PLA, PGA, and PLGA are used for scaffold manufacturing to restore the function of damaged organs or tissues. The FDA has already approved PLA and PGA uses for different medical implants due to the safe elimination process of lactic and glycolic acid secondary products [166]. Jia et al. [97] developed a scaffold for oral bone defect restoration. Three-dimensional composite scaffolds made of PLGA and superparamagnetic iron oxide nanoparticle coatings were implanted in rat animal models to analyze the palate–bone regenerating effects and their interaction with the oral microbiota. These special MNP-coated implants induced an excellent bone regeneration effect. Regarding oral bacteria, a decrease in the *Clostridium* spp. population and a dominant flora consisting of *Proteobacteria* were put in evidence. Although MNPs had a beneficial effect on bone regeneration, they altered the oral microbiota in rats. MNPs upregulated hepcidin and the concentration of iron serum.

## 3. Structural and Morphological Aspects of Polymeric Scaffolds Loaded with MNPs

**Figure 6:**
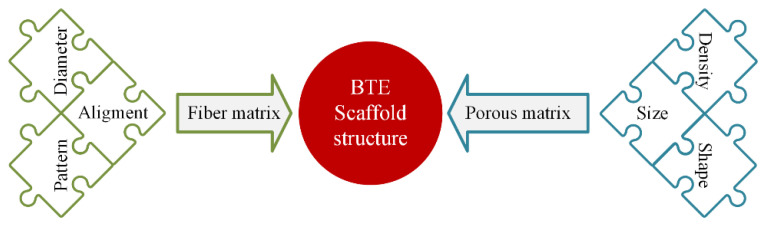
The most important structural and morphological parameters of a scaffold used in BTE.

In scaffold design for the BTE domain, different structural parameters should be considered because the implant must mimic the ECM of the tissue, which has to be replaced. The cellular response is strongly influenced by parameter modification [167]. As mentioned in the Introduction section, surface roughness, wettability, and pore scaffold characteristics such as shape, size, and density are directly linked to cell differentiation, proliferation, and gene expression. Another main parameter that influences the structural and morphological aspects of the scaffolds is the fiber diameter and alignment. The main structural and morphological scaffold parameters that affect the cell behavior are presented in Figure 6.

**Figure 7:**
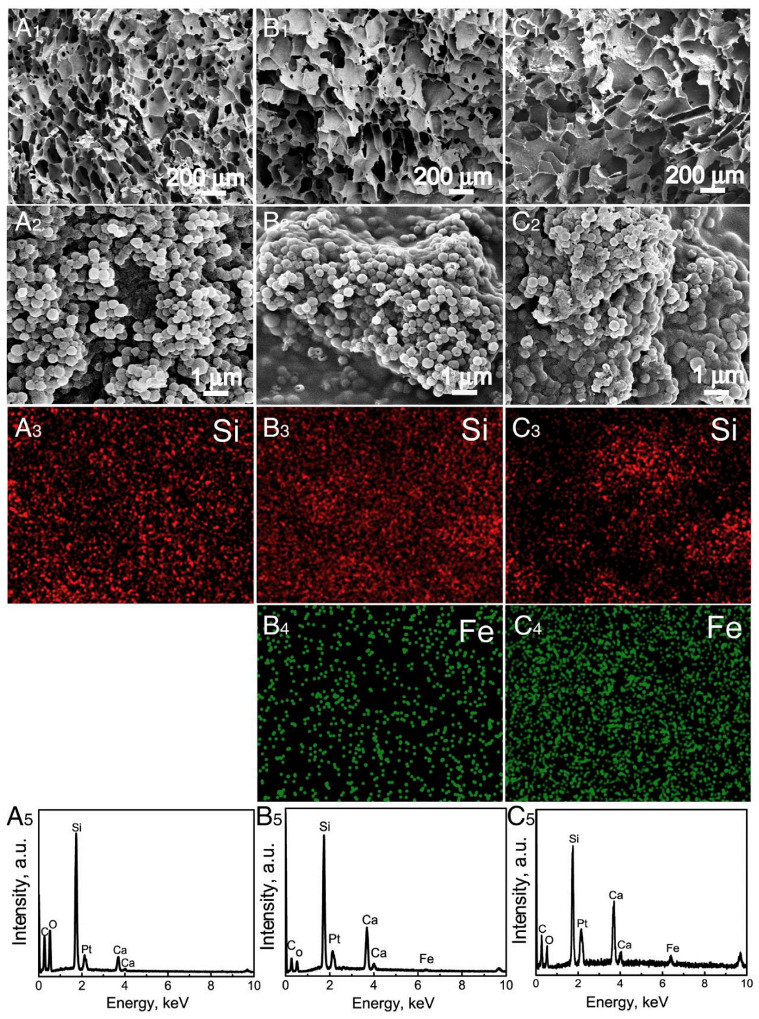
SEM investigation, silicon and iron distribution, and energy-dispersive X-ray spectroscopy for scaffolds prepared using freeze-drying technology: (**A**_1_–**A**_5_) [CS/MBG] images and (**B**_1_–**C**_5_) [CS/MBG]/[SrFe_12_O_19_] images [86]. Reprinted from [86] Copyright (2023), with permission from Elsevier.

**Figure 8:**
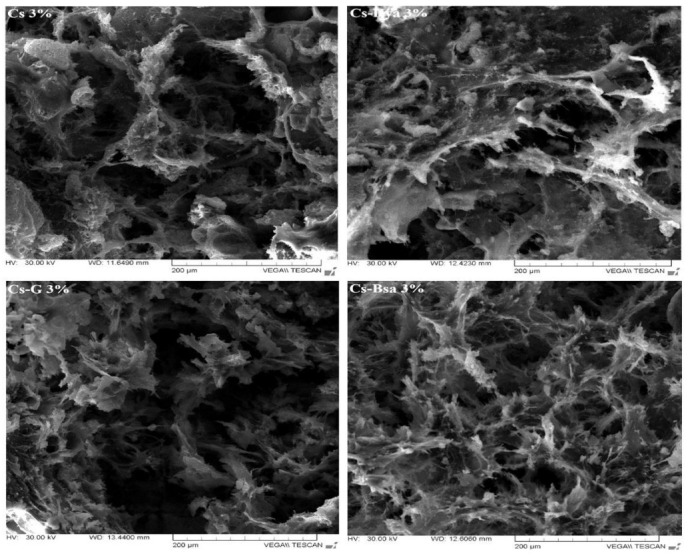
SEM images obtained for scaffolds made from [CS], [CS/HyA], [CS/G], and [CS/BSA] with a concentration of 3% MNPs [87]. Reprinted from [87], Copyright (2023), with permission from Elsevier.

Zhao et al. [85] prepared a composite matrix from CS/Col in which they introduced nanohydroxyapatite and magnetite nanoparticles. The manufactured scaffolds were characterized by high porosity with interconnected pores with sizes between 100 and 300 μm. The authors concluded that the implant structure and surface roughness were similar to those of human bone and facilitated the proliferation and adhesion of cells and the circulations of nutrients. Lu et al. [86] developed composite porous magnetic scaffolds for cancer treatment and bone regeneration. A mixed solution containing CS, MBG microspheres, and SrFe_12_O_19_ magnetic nanoparticles was prepared. Finally, 3D scaffolds characterized by interconnected macropores with an average size of 200 μm were obtained (Figure 7). Cojocaru et al. [87] carefully investigated the porosity of composite magnetic scaffolds based on innovative biopolymer combinations prepared using a biomimetic coprecipitation method. They found that the implant porosity varied as a function of polymeric matrix composition, and the average pore size was about 994 μm for the CS 3% scaffold and 1115.25 μm for the CS-HyA 3% implant (Figure 8). As an overall conclusion, all the scaffolds were characterized by a 3D structure with interconnected pores and included calcium phosphate and MNPs. Dashnyam et al. [88] made hybrid magnetic scaffolds based on gelatin–siloxane with interconnected microporous morphology and uniform distribution of pores. The MNPs were homogeneously distributed in the polymeric matrix, and no particle agglomerations were present. Samal et al. [89] produced biomimetic magnetic silk scaffolds and noticed that the morphology of the implants was not affected by the magnetization process. The pore size was almost the same in the case of the silk implant and the magnetic scaffold. Very little aggregation of MNPs as clusters of 50–200 nm was microscopically visualized, and it was concluded that the silk interacted very well with the magnetic nanoparticles, and homogenous biomaterial adequate for bone regeneration and tumor treatment was developed. Ganesh et al. [90] developed PCL-based nanofibrous scaffolds doped with nanohydroxyapatite and gadolinium particles. Regarding the scaffold morphology, randomly oriented nanofibers with a diameter between 100 and 500 nm were put in evidence, and it was concluded that the fiber diameter decreased due to the inclusion of HAp and gadolinium particles. The sample wettability was tested using water at 25 °C and 65% humidity. It was noticed that the contact angle decreased from 146° for the PCL scaffold to 130° for the composite PCL-based scaffold. Dankova et al. [91] made 2D poly-ε-caprolactone nanofibers incorporating MNPs. A nano/microfibrous morphology was put in evidence. A dominant nanofibrous fraction with a mean fiber diameter of 216 nm and a microfibrous fraction with a mean diameter of 1138 nm were components of the scaffold mesh. Images obtained through scanning electron microscopy showed that an important quantity of MNPs was placed inside the polymeric fibers.

De Santis et al. [92] fabricated 3D composite implants from a PCL matrix in which iron hydroxyapatite particles were inserted. Based on scanning electron microscopy combined with energy-dispersive spectroscopy (SEM/EDS), aggregates of FeHAp uniformly distributed in the matrix were put in evidence. Transmission electron microscopy showed an amorphous calcium phosphate matrix with Fe particles. The calcium phosphate particles exhibited a needle shape, and their sizes ranged between 5 and 20 nm in width and between 50 and 80 nm in length. Kim et al. [93] made magnetic scaffolds based on PCL with an average porosity of 74.6% for 5% wt. MNPs and 70.9% for 10% wt. MNPs. It was noticed that since the porosity percentage was almost the same for all the scaffolds, the density level increased directly proportionally to the MNP concentration. Using X-ray diffraction (XRD) investigations, characteristic peaks for magnetite were observed, and an average particle size of about 10.7 nm was computed using the Scherrer equation. The addition of MNPs resulted in a decrease in the contact angle from 85° for pure PCL scaffold to 61° measured in the case of 5% wt. MNPs and 47° for 10% wt. MNPs. Safari et al. [168] manufactured biofunctional phosphorylated polycaprolactone combined with a gelatin magnetic scaffold. SEM investigations showed that all the scaffolds exhibited a 3D porous structure with interconnected and open macropores. The average pore size for the PCL/G samples containing MNPs was about 240 μm. It was noticed that the addition of MNPs resulted in a decrease in the porosity percentage of the implant. Singh et al. [140] investigated the potential of magnetic nanofibrous scaffolds of poly(caprolactone). Transmission electron microscopy and XRD characterization point out an average size for the MNPs of 11 nm. Nanofibrous scaffolds were made through electrospinning for different MNP concentrations. Microscopy images put in evidence continuous and smooth fibers with different average diameters as a function of MNP concentration. For 5% wt. MNPs, the measured fiber diameter was about 864 nm, and in the case of 15% wt., the MNP diameter decreased to an average value of 200 nm. The apparent contact angle decreased directly proportionally to the MNP increase. For the 10% wt. MNPs, it was measured at 68°, which decreased to 47° in the case of 20% wt. MNPs.

Structural and morphological aspects of polymeric scaffolds loaded with MNPs cannot be uniquely established for all cellular responses. To promote individual cell proliferation and adhesion, the scaffold design must be adapted as a function of targeted application characteristics.

## 4. Properties of Polymeric Scaffolds Loaded with MNPs

The most important properties of polymeric scaffolds reinforced with MNPs are mechanical and thermal. The implant must present adequate stability to substitute the missing hard or soft tissue and can be useful in tumor treatment as required.

Many literature studies have provided valuable information regarding topography, morphology, the existence of adhesion sites for living cells, and mechanical properties, putting in evidence that the abovementioned factors are significant with respect to scaffold integration inside the human body [169,170,171]. The most investigated mechanical properties are tensile strength, Young’s modulus, and fracture toughness because they impact cell proliferation [172]. It is of great interest to develop a scaffold that sustains a proper mechanical microenvironment favorable to a physiological medium, which determines cell development [173]. Biomechanical signals emitted by cells are linked to increased stem cell differentiation for implants with rough surfaces [174]. Applying mechanical forces can guide the differentiation and proliferation of the cells, leading to tissue formation under well-controlled conditions. The ECM is important because it facilitates cell viability through biochemical interactions such as adhesive motifs and growth factors and mechanical characteristics such as stiffness and deformability. Vogel and Sheetz [175] and Wang et al. [176] proved that mechanical signals significantly impact cell proliferation, adhesion, and death. Studies regarding the mechanical properties of natural and synthetic polymeric scaffolds loaded with MNPs are summarized in Table 4.

Even if the implant suffers a decrease in mechanical properties due to its degradation in the biological medium, the cells can strengthen the scaffold body because they produce ECM and reconstruct the surrounding tissue. Different physiological loads act on an implanted scaffold, dependent on cellular traction forces and/or host tissue, resulting in tissue deformation near the implantation site. Traction forces appear during the cell attachment process, while in cell seeding, scaffold contraction manifests. The implant stiffness must have similar values to those of host tissue, and the scaffold elasticity has to be adequate to absorb the forces due to cell movements [177].

**Figure 9:**
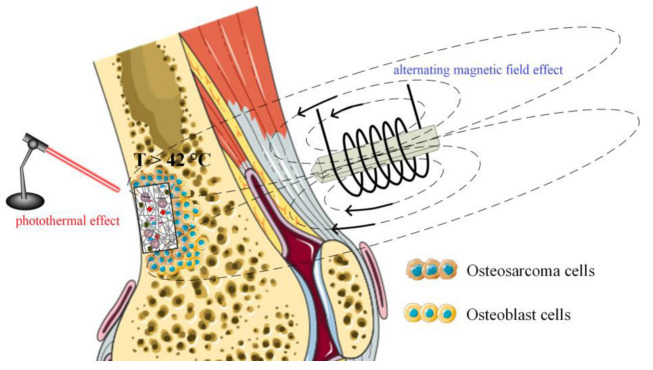
Magnetic hyperthermia and photothermal therapy treatment for oncological disease in BTE. The figure was generated using images assembled from Server Medical Art, which are licensed under a Creative Commons Attribution 3.0 unported license (https://smart.servier.com, accessed on 9 January 2023).

Scaffold thermal properties are fundamental in oncological tumor treatment. Based on MNP properties through hyperthermia treatment (HT), which consists of a local temperature increase above 42 °C for a duration between 30 and 60 min, the deoxyribonucleic acid of cells is damaged [178]. During the HT process, cellular protein denaturation, extracellular pH increase, and free radical apparition of can be reported [179]. Magnetic hyperthermia enhances the body’s immunomodulation through the release of heat-shock protein. Usually, MNPs incorporated into scaffold material exposed to an alternating magnetic field through magnetocaloric effect transform magnetic energy into heat, which is released in the implant vicinity. The scaffold must be adequate for tumor-related bone defect treatment, and it also has the ability to eliminate malignant tumor recurrence due to residual cell existence. Another treatment of oncological pathologies consists of photothermal therapies, which involve materials containing MNPs. It was observed that under the effect of near-infrared light (NIR), the temperature can increase up to 42–50 °C, which permits tumor hyperthermia ablation. The bifunctional character of magnetic scaffolds, including the bone regeneration process combined with systematic MNP treatment, such as bone-targeting nanoparticles for tumors, is depicted in Figure 9.

Lu et al. [86] incorporated M-type hexagonal ferrites (SrFe_12_O_19_) into an MBG/CS polymeric matrix. They applied NIR conditions and investigated the effect of MNP-based implants on the MG63 cell line. After 6 min of irradiation, a temperature increase of about 45 °C was attained. Results consisting of osteosarcoma cell death were reported in the case of scaffolds containing MNPs. The authors also conducted *in vivo* tests and proved increased necrosis of about 84.6% in the tumor region. They concluded that SrFe_12_O_19_ nanoparticles have a strong antitumoral effect when NIR light is applied.

In [89], through an infusion technique, silk scaffolds with low (50 μL/mL) and high concentrations (250 μL/mL) of MNPs were developed. The application of an external magnetic field permitted the evaluation of the thermal response of implants. It was concluded that because the scaffolds exhibited a low saturation magnetization value, they were characterized by good magnetic hyperthermia properties, producing an increase in temperature of about 8 °C above the 37 °C level. Zhang et al. [95] investigated the magnetic hyperthermia properties of composite MBG/PCL scaffolds dopped with Fe_3_O_4_ magnetic superparamagnetic nanoparticles. It was noticed that the samples with 5% wt., 10% wt., and 15% wt. SPIONs exhibited an increase in temperature when an alternating magnetic field with a maximum value of magnetic flux density of 180 Gs and a frequency of 409 kHz was applied. For 15% wt. SPIONs, the temperature increased from 20 °C to 43 °C in 2 min. The specific absorption rate (SAR) index for the scaffolds had values between 1.4 W/g for 5% SPIONs and 4.7 W/g measured in the case of 15% wt. SPION implants. Lodi et al. [139] analyzed “if and how” the MNP concentration affected the hyperthermia treatment of residual cancer cells. They developed a non-linear multiphysics problem in which they set the magnetic induction at 30 mT and a frequency to 300 kHz. By choosing two selected loading values, they concluded that the scaffolds exhibited well-defined behavior when the temperature increased. The main finding of this simulation was that different therapeutic results could be estimated and investigated through a clear visualization of the material temperature patterns, resulting in a high dependence on the MNPs’ loading characteristics and condition.

Espinosa et al. [180] investigated the duality of iron oxide nanoparticles in cancer treatment. They observed the amplification of the heating effect through magnetic hyperthermia combined with photothermal therapy. Iron oxide nanocube suspension was used to measure the magnetic hyperthermia effect on three cancer cell lines, i.e., PC3 (prostate cancer), SKOV3 (ovarian cancer), and A431 (epidermoid cancer). The magnetic field strength varied between 5 and 24 kA/m, and the frequency ranged from 320 kHz to 1.1 MHz. This condition was combined with the effect of laser hyperthermia induced by an NIR continuous laser at 808 nm. For the *in vivo* tests, 22 female Naval Medical Research Institute (NMRI) immunodeficient nude mice were involved, and different solid tumors were artificially created by injecting human cancer cells. A complete tumor remission was noticed in the case of all animal models when the bimodal treatment was applied. Future research in the magnetic scaffold domain must include these two thermal properties of MNPs to achieve fast and efficient treatment of different oncological pathologies.

## 5. Magnetic Nanoparticle Content and Magnetic Properties of Scaffolds Loaded with MNPs

**Figure 10:**
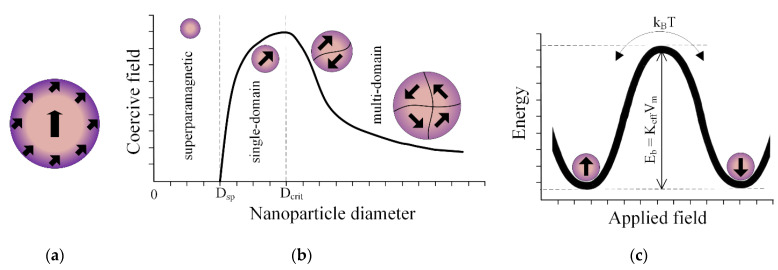
Magnetization processes in MNPs: (**a**) illustration of surface spin-canting effect in MNPs; (**b**) coercive field dependence as a function of nanoparticle diameter; (**c**) nanoparticle energy versus external applied magnetic field.

MNPs are characterized by unique properties such as high surface-to-volume ratio and magnetic responses, which are influenced by small particle diameters and differ from bulk materials. Two physical quantities are the most important part of MNP use in biomedical therapies. A higher magnetic moment is helpful in magnetic imaging and biosensing applications, while a higher magnetic field strength value is sought in the case of the magnetic theragnostic domain [181]. It was observed in [182,183,184,185] that a surface spin-canting effect, which determines the differentiation of magnetic properties between the surface layer and the core of MNPs, can generate a decrease in saturation magnetization followed simultaneously by an increase in the anisotropy constant (Figure 10a). This fact is considered the opposite of the bulk material magnetic phenomenon. In the case of a spherical magnetic nanoparticle, the saturation magnetization (M_s_) can be expressed as a function of magnetic core diameter (D), the thickness of the spin-canting layer (δ), and the saturation magnetization of the bulk material (M_sb_) (Equation (1)).

M_s_ = M_sb_ (1 − 2δ/D)3. (1)

For the effective anisotropy constant, in [186] K_eff_ was determined to depend on the bulk (K_b_) and surface (K_s_) anisotropy constants through a shape parameter (Φ) and magnetic core diameter (D) (Equation (2)):

K_eff_ = K_b_ + (6Φ/D)K_s._ (2)

The magnetization processes in the case of MNPs depend on the particle diameter, so a critical value (D_crit_) can be considered a boundary between the single magnetic domain state and the multidomain configuration (Figure 10b) [77]. The highest magnetic moment value characterizes single-domain MNPs because the particle magnetization vector is oriented in only one direction and equal to saturation magnetization. Stoner-Wohlfarth’s model [187] considered that the magnetization of a monodomain particle rotates as if it were only one giant magnetic moment. This phenomenon is called “macro-spin approximation”. Superparamagnetic behavior occurs in the case of small-diameter ferro- or ferrimagnetic nanoparticles. It is well known that in the case of a single-domain particle, there are two antiparallel preferred orientations of the magnetic moment along the easy magnetization axis. Between these directions is an energy barrier (E_b_), which prevents the switch of the magnetic moment from one stable equilibrium position to the other minimum-energy state (Figure 10c). Another critical particle diameter size (D_sp_) at which it is possible to transition from the monodomain state to superparamagnetic behavior must be considered. If this geometrical dimension is reached at a given temperature at which the energy barrier becomes comparable to thermal energy (k_B_T, where k_B_ is the Boltzmann constant), the magnetic moment flips from one preferred direction to another. The fast reversal of the magnetic moments exhibits a null magnetic moment without an externally applied magnetic field. In opposition, the magnetic moments align along the external magnetic field, so as a consequence, a net magnetization value is attained. The particles present an anhysteretic behavior when different magnetic field values are considered. The magnetization curve has a reversible S shape that the Langevin model can approximate according to Equation (3) as follows [188]:

M(H) = M_s_L[μ_0_μH/(k_B_T)], (3)

where L(x) = coth(x) − 1/x represents the Langevin function, μ_0_ is the vacuum magnetic permeability, H is the magnetic field strength, and μ is the absolute value of the particle magnetic permeability.

Superparamagnetic nanoparticles are usually included in polymeric scaffolds for tissue engineering due to their stability over time and their increased biocompatibility. Lu et al. [86] analyzed the influence of MNP content on the magnetic properties of SrFe_12_O_19_ nanoparticles incorporated into MBG/CS porous scaffolds. They developed two samples with SrFe_12_O_19_ masses of 0.125 g (MBCS1:7) and 0.25 g (MBCS1:3) and MBG masses of 0.875 g and 0.75 g. These mixtures were added to CS solutions. The saturation magnetization depended on a high MNP content, so for the MBCS1:7 sample, it was measured at a value of 4.44 emu/g, and in the case of MBCS1:3, a value of 7.68 emu/g was obtained. The experimental coercivities were found to be 4120 Oe and 5102 Oe, respectively. It can be noticed that this particular type of M-ferrite nanoparticle exhibited a hysteresis cycle that showed that they are not in a superparamagnetic state. Cojocaru et al. [87] investigated the magnetic properties of different polymeric matrices (CS, CS-HyA, and CS-BSA), in which they integrated 1% wt., 3% wt., and 5% wt., respectively, of Fe_3_O_4_ nanoparticles coated with CS. For the 5%wt MNPs, the magnetization was found to be equal to 10.14 emu/g (CS), 12.53 emu/g (CS-HyA), and 8.16 emu/g (CS-BSA). Dashnyam et al. [88] reinforced hybrid porous scaffolds from GS with Fe_3_O_4_ up to 3% wt. The magnetic properties were investigated using superconducting quantum interference device (SQUID) magnetometry, and the MNPs showed typical superparamagnetic behavior with S-shaped magnetization curves. The saturation magnetization increases proportionally with the MNP content from 0.24 emu/g (1% wt. MNPs) to 0.64 emu/g (3% wt. MNPs). Samal et al. [89] determined the magnetic properties of silk scaffolds in which they diffused 50 μL/mL or 250 μL/mL MNPs. The magnetic measurements were performed at 37°, and a superparamagnetic-like response characterized by saturation magnetization values of 2.7 emu/g and 13 emu/g was obtained. The coercive field was about 15 Oe, a value considered negligible. In [91], PCL scaffolds with MNPs (γ-Fe_2_O_3_) were prepared through the electrospinning method. The magnetization curves showed a typical trend for iron oxide nanoparticles with a diameter above 20 nm. The saturation magnetization of the samples was found to be about 6.1 Am^2^ at 300 K, with an estimated content of MNPs of 7.9% wt. Kim et al. [93] prepared magnetic scaffolds from PCL and MNPs with contents of 5% wt. and 10% wt., respectively. Experimentally determined saturation magnetizations of 1.6 emu/g and 3.1 emu/g were obtained. The coercivity and remanence points were impossible to determine, so the superparamagnetic state of the MNPs was confirmed. It was noticed that 80% magnetic saturation was attained at 0.5 kOe in a linear field dependence. Zhang et al. [95] developed composite magnetic scaffolds with a matrix of MBG/PCL polymers through additive manufacturing technology. They inserted Fe_3_O_4_ nanoparticles in 5% wt., 10% wt., and 15% wt. The saturation magnetization was between 1.01 emu/g (5% wt. MNPs) and 2.90 emu/g (15% wt. MNPs). Shuai et al. [96] manufactured PLLA/PGA scaffolds that incorporated Fe_3_O_4_ MNPs with 2.5% wt., 5% wt., 7.5% wt., and 10% wt. through the SLS method. According to vibrating sample magnetometry (VSM), the samples showed no measurable coercive field and remanent induction values for each MNP percent. A saturation magnetization value proportional to the MNP content was obtained, starting with 1.66 emu/g to 8.51 emu/g. Singh et al. [140] made PCL nanofibrous scaffolds incorporating 12 nm diameter Fe_3_O_4_ in concentrations of 5% wt., 10% wt., and 20% wt. It was concluded that the saturation magnetization increased from 1 emu/g to 11.2 emu/g relative to the mass fraction of MNPs placed into the polymer matrix. The specific hysteresis energy losses at the maximum applied field of 20 kOe were estimated to be 2.4 × 10^3^ erg/g (5% wt. MNPs), 5.5 × 10^3^ erg/g (10% wt. MNPs), 12.5 × 10^3^ erg/g (15% wt. MNPs), and 22.3 × 10^3^ erg/g (20% wt. MNPs).

The MNP percentage in scaffolds can have an important influence on the therapeutic properties of implants in osteogenesis and cancer treatments. Lodi et al. [139] showed that spatial loading influenced the saturation magnetization of the samples in a direct manner. The magnetic properties of scaffolds can be tuned as a function of the preparation method, and magnetic cluster formation must be avoided because non-linear magnetic effects appear and negatively influence the treatment. Including MNPs in biodegradable polymeric matrices is of great importance because innovative therapies strongly dependent on a well-established percentage of MNPs can be applied in clinical applications.

## 6. Magnetic Field Effects on Biocompatibility and Osteogenesis

MNPs have proven to be a very efficient tool in tissue engineering due to the fact that they produce an intense cell induction effect by generating an intrinsic magnetic field [189,190]. Nanoparticles are internalized by cells, so as a consequence, activation of intracellular pathways that enhance osteogenesis can occur [71,191]. When MNPs or SPIONs are reinforced inside a scaffold, the resulting magnetization sustains the substance changes that occur between receptors placed on the cell membrane and ion channels, which is directly linked to improved osteogenic proliferation and differentiation [76]. Cell adhesion is promoted, and the mechanical properties of the scaffold are improved, as shown in Section 4. MNPs are essential in angiogenesis when an external magnetic field is applied. Under the effect of an alternating magnetic field, MNPs can transport different drugs or even mesenchymal stem cells (MSCs) to a bone defect site [192].

**Figure 11:**
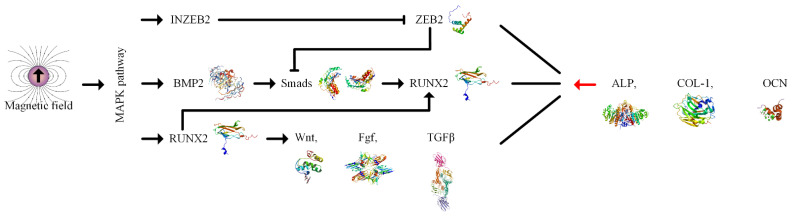
The influence of the magnetic fields of MNPs/SPIONs on the osteogenic differentiation of stem cells in accordance with the mitogen-activated protein kinase (MAPK) pathway.

In the case of MSCs, magnetic stimuli can be recognized through the cytoskeleton or membrane of the cells that transmit chromosomal responses with a significant influence on gene expression and protein synthesis [193,194]. SPIONs can provide mechanical stimulation to the membrane of MSCs; furthermore, the intrinsic magnetic field of the particle can act on the mitogen-activated protein kinase (MAPK) pathway, even in the absence of an external magnetic field [195,196]. This phenomenon determines an overexpression of runt-related transcription factor (RUNX2), defined as an early osteogenesis differentiation marker, and an upregulation of bone morphogenetic protein 2 (BMP2), which activates the Smads proteins. These types of proteins are the principal signal transducers involved in the transforming growth factor β (TGFβ) receptors and enhance the expression of RUNX2 [197]. The upregulation of INZEB2, which is of high importance in the osteogenesis process, has a direct consequence in the downregulation of zinc finger transcription factor 2 (ZEB2), which suppresses the BMP2/Smads/RUNX2 pathway [137,198]. As a final result, alkaline phosphatase (ALP), osteocalcin expression, and collagen type I (COL-1) increase and have a positive impact on the osteogenesis process (Figure 11).

Safari et al. [168] tested the biocompatibility capabilities of biofunctional phosphorylated magnetic scaffolds for BTE. They used human dental pulp stem cells (hDPSCs) seeded at 37 °C in 96-well plates at a density of 5 × 10^3^ cells/well. The MNP effect consisted of increased ALP activity and higher expression level of RUNX2 and BMP2 osteogenetic biomarkers. The use of phosphorylated polycaprolactone determined a very good implant osteoconductivity due to the upregulation of COL1, RUNX2, BMP2, and osteocalcin (OCN) genes. They concluded that the developed scaffolds exhibited high biocompatibility. In the case of magnetic nanofiber scaffolds [140], MSCs derived from rat bone marrow were used to analyze osteogenesis. It was noticed that osteoblastic cell adhesion was amplified by the MNPs, and good penetration through the implant nanofibers was put in evidence. To analyze osteoblastic differentiation, the ALP activity was determined after cell culture for 7 and 14 days. By adding samples to the ALP reaction medium, an upregulation of this parameter was observed. This result was supported by analysis of the expression of mRNA levels of bone-associated genes such as COL1, osteopontin (OPN), and bone sialoprotein (BSP). Biocompatibility investigations proved that a magnetic nanofiber implant is an excellent candidate for BTE. Another approach in scaffold manufacturing based on additive manufacturing technology was presented in [96]. The magnetic microenvironment generated by PLLA/PGA/MNPs 3D scaffolds exhibited good biocompatibility in the case of MG63 cells. The cells were incubated in Dulbecco’s modified eagle medium (DMEM) supplemented with sodium pyruvate, 10% fetal bovine serum (FBS), and antibiotics under standard testing conditions. The sterilized magnetic scaffolds were placed in 24-well culture plates, and 4 × 10^5^ MG63 cells were used for each scaffold. Good cell adhesion was evidenced through SEM investigations, and high cell viability was obtained after a cell counting kit-8 (CCK-8) assay was performed. The qualitative staining and quantitative activity of ALP showed high osteoblastic differentiation of cells placed in the scaffold vicinity. Zhang et al. [95] tested the biocompatibility of other 3D-printed magnetic composite scaffolds (Fe_3_O_4_/MBG/PCL) on hBMSCs. It was noticed that integration of MNPs into a polymeric composite matrix had no significant effect on the mineralization ability of the implant but upregulated the ALP activity and the osteogenesis-related gene expression (OCN, RUNX2, BSP, BMP2, and COL-1), putting in evidence an increased osteogenesis effect. In the case of the 15% wt. MNPs scaffold, the determined values of osteogenic expression were found to be almost double those for the 5% wt. and 10% wt. MNPs. Chen et al. [94] prepared a magnetic cell–scaffold interface by incorporating SPIONs. The scaffolds’ biocompatibility was investigated on Spraque–Dawley rat adipose-derived mesenchymal stem cells (ADSCs). Using confocal laser scanning microscopy, cell morphology was visualized at 6 and 24 h after cell adhesion. The viability was measured at 1, 4, 7, and 10 days with the help of a CCK-8 kit. It was concluded that the highest cell viability and adherence were obtained in the case of MNP-containing scaffolds. ALP activity analysis was used to evaluate osteogenic differentiation. The magnetic scaffolds presented the highest values of this indicator, proving an intense osteogenic process. Dankova et al. [91] presented a practical approach to *in vitro* MSC proliferation based on PCL/MNP nanofibrous scaffolds. The MSCs were extracted from the ilium bone marrow of miniature pigs and sterilized at 37° by ethylene oxide. The cells were seeded on scaffolds in 96-well plates at a density of 63 × 10^3^/cm^2^. The cell proliferation, as well as metabolic and ALP activities, as observed for 21 days. Based on the MTS assay, it was concluded that the cell metabolic activity and viability were improved when MNPs were added. Regarding the ALP activity, a significant increase was put in evidence in the case of MSCs cultivated on magnetic scaffolds on days 7 and 21. Cojocaru et al. [117] investigated the biocompatibility of microporous biomimetic scaffolds loaded with MNPs. They used sarcoma osteogenic (SaOS-2) cells and performed all measurements following ISO 10993 standard [199] by quantifying the cell viability through lactate dehydrogenase (LDH) release. It was concluded that because they developed a very complex implant with different MNP concentrations, the cell behavior and viability were strongly influenced by various parameters such as particle diameter and shape, the chemical composition of the scaffold matrix, and implant morphologic and magnetic properties. Lu et al. [86] incorporated M-type ferrite nanoparticles in MBG/CS porous scaffolds. The hBMSC cell line was used to investigate the implant biocompatibility. Well-spread cell morphology was observed in the case of MNP-based scaffolds. They concluded that SrFe_12_O_19_ nanoparticles are highly biocompatible and that the scaffolds promoted cell proliferation and adhesion. Osteogenic differentiation was analyzed using real-time polymerase chain reaction (PCR) and Western blotting. The highest level of osteogenic gene expression was found in the case of a higher percentage of MNPs. Table 5 provides examples of biological response (i.e., cell viability and proliferation and bone markers) for polymeric magnetic scaffolds.

In the case of an external magnetic field presence, the magnetic stimulation applied by the MNPs to the cells is enhanced, so osteogenesis and angiogenesis are improved [70,200]. It was noticed that an electromagnetic field (EMF) might lead to a high amount of cell migration, adhesion, and differentiation, as a direct consequence of which the tissue regenerates faster than in the absence of an EMF [201,202]. The combination of magnetic polymeric scaffolds for BTE and EMF exposure has recently received considerable scientific interest; some such studies are presented in Table 6 [69,203,204,205,206].

It can be noticed that the existence of an external electromagnetic field produces beneficial effects on angiogenesis and osteogenesis, but it adds many complications regarding the type of devices that can be clinically employed. The devices must be easy to use, adequate for specific patient anatomy, with facile follow-up, and preferably with low-cost EMF-generating components. More research is needed to determine whether the application of an external EMF is necessary in the case of magnetic scaffolds because MNPs with sufficiently strong magnetization that sustains increased osteogenic and angiogenic effects can be developed.

Magnetic nanoparticles can be internalized by stem cells because they promote osteogenic differentiation. The percentage of MNPs is an important parameter in biomedical applications because they tend to accumulate in the kidney, bone marrow, spleen, and liver [210]. The macrophage cells in the reticuloendothelial system (RES) internalize MNPs with reduced diameter that are subjected to acid-induced degradation [211,212]. Numerous investigations involving *in vitro* and *in vivo* studies have revealed that the toxicity is dose-dependent and that the maximum quantity of MNPs should be chosen by taking into account the type of nanoparticles and living cells [213,214,215,216,217]. Free ionic iron, which results from the degradation of iron oxide nanoparticles, is stored in ferritin protein and is used in normal cellular functions. Another transmembrane protein is ferroportin, which is involved in iron export from cells, from where it is transported into the bloodstream through transferrin [218]. Usually, cells control the level of ferritin and ferroportin and maintain a balance between stored and exported ionic iron simultaneously with the downregulation of transferrin receptors, which limit the assimilation of iron from the blood [218]. The toxic effect of MNPs appears when free ionic iron that remains unbound takes part in a Fenton reaction, resulting in the appearance of reactive oxygen species (ROS) [77,219]. Fan et al. [220] noticed that in the case of an intracellular iron content of about 13 pg, the osteogenesis process decreased for BMSCs labeled with citric-acid-coated SPIONs. They concluded that this high iron concentration led to ROS species formation, which reduced cell viability. In another study conducted by Andreas et al. [221] using the same type of SPIONs, an intracellular iron content of 70 pg was reported, which did not have an important influence on human stem cells. Animal stem cells did not support a higher grade of MNP toxicity compared to human cell lines. When MNPs are incorporated into scaffolds, their toxicity grade is reduced. All cytotoxicity analyses must be conducted in accordance with the ISO 10993 standard, which states that a material exhibits non-cytotoxic effects in the case of cell viability higher than 70% in comparison with control samples [199]. Cojocaru et al. [117] analyzed the cytotoxicity of a magnetic polymeric scaffold by quantifying the LDH release. They found that on day 7 in some culture plates, the LDH release decreased below 60%, indicating that the samples with 50% COL, 50% CS, and Ca/P between 1.57 and 1.72, and 5%wt. MNPs exerted important cytotoxicity over the SaOS-2 cells. In [87], magnetic scaffolds with a natural polymer-based matrix and 1% wt., 3% wt., and 5%wt. MNPs showed cell viability higher than 96%, indicating that the increased MNP percentage did not result in significant toxicity. A [CS/BSA]/[3% wt. Fe_3_O_4_] scaffold was chosen for a live/dead staining assay performed for fibroblasts. It was noticed that after 96 h of incubation, the cell viability increased, proving that this MNP concentration is suitable for BTE. Sometimes, MNPs form aggregates that can impact cell adhesion and proliferation. De Santis et al. [92] made PCL/FeHAp scaffolds in which they seeded BMSCs that were magnetically labeled with MNPs in a concentration between 1.04 and 8.33 μg/mL. An MNP/cell ratio of 16.6 μg/1000 cells was set for the cytotoxicity tests. They concluded that in the case of higher MNP loading, the number of cells in the scaffolds was reduced due to magnetic agglomerate formation. In [168], [PCL/G]/[MNPs] scaffolds were prepared, and due to the hydrophilic character of the MNPs, hDPSC growth and adhesion properties improved. This fact can be attributed to the phenomenon whereby, through the intrinsic peroxidase-like activity of the MNPs, a diminution of intracellular H_2_O_2_ occurred, and accelerated cell cycle activity was induced [222].

Most of the presented studies put in evidence that MNPs reinforced into polymeric scaffolds do not exhibit toxic effects against the cell. Still, implant biocompatibility and cytotoxicity analysis must be carefully considered before introducing such scaffolds in animal testing or clinical trials.

## 7. Animal Testing

**Figure 12:**
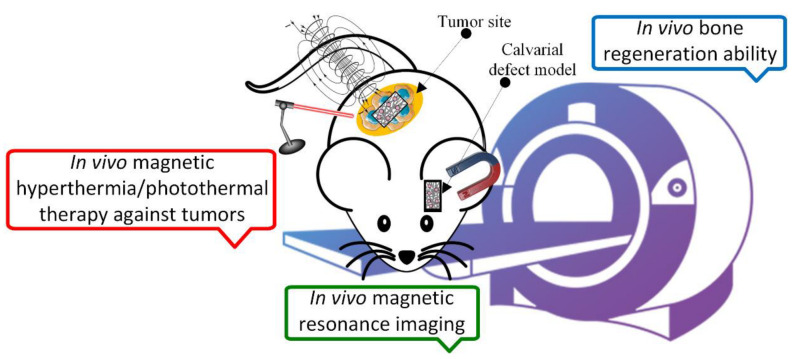
Representation of the main function of the magnetic scaffold applied for *in vivo* tests.

Scaffolds must be biologically compatible with the animal model tissue to permit proliferation, adhesion, and differentiation of the host cells. As explained in Section 6, the biocompatibility and toxicity of the implant must first be investigated *in vitro*, and if the safety requirements are met, it can be implanted in the animal body. Figure 12 shows a schematical representation of the main functions of magnetic scaffolds regarding *in vivo* studies that involve rodents.

**Figure 13:**
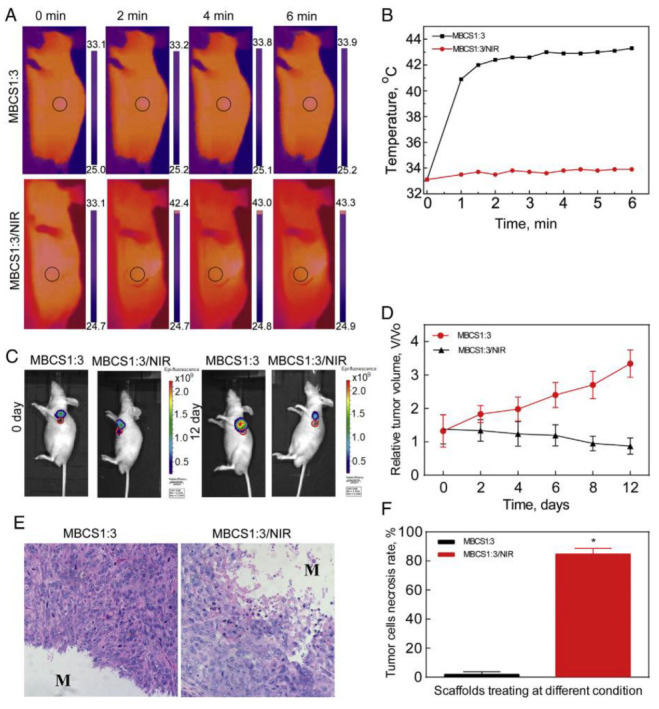
*In vivo* photothermal therapy: (**A**) infrared thermal images; (**B**) heating curves of scaffold tumor-bearing mice in the presence/absence of an NIR laser; (**C**) rodent body fluorescence images with or without NIR laser irradiation; (**D**) relative tumor volume evolution over time; (**E**) H&E stained images of tumor tissue; (**F**) tumor cell necrosis rate (* *p* < 0.05 versus control) [86]. Reprinted from [86], Copyright (2023), with permission from Elsevier.

Zhao et al. [85] used male SD rats weighing 300 g to investigate the *in vivo* behavior of magnetic [CS/Col/nHAp] scaffolds reinforced with Fe_3_O_4_ nanoparticles. They induced an osseous defect with a 5 mm diameter on the middle ridge of the rat skull. Sample scaffolds with an area of 5 × 1 mm^2^ were implanted inside the defect of some animals. For the analysis, a control group was considered, including rats with a bone defect in the skull and no implant use. Post-surgical antibiotic treatment was provided, and no complications were reported. All the animals were euthanized after 12 weeks, and microcomputed tomography tests were conducted to evaluate bone growth in the calvarium, with histological assessment to investigate the histological repair process. The group with magnetic scaffolds exhibited good osteointegration and a gradual implant degradation that supported new bone formation. The inclusion of MNPs in the polymeric matrix favored a magnetocaloric effect, which produced dynamic bone growth. It was concluded that the best osteogenesis phenomenon was observed when magnetic properties were added to the polymeric scaffold. Lu et al. [86] investigated the bone regeneration properties and photothermal therapy efficiency of [MBG/CS]/[SrFe_12_O_19_] scaffolds implanted in 12 male SD rats with an average weight of 310 g. Bilateral calvarial defects were made, and implants with a thickness of 2 mm and a diameter of 5 mm were inserted. All the animals were injected with fluorescent dye under general anesthesia conditions at 2, 4, and 6 weeks to observe new bone formation at an interval of 12 weeks. After micro-CT investigations, it was proven that SrFe_12_O_19_ resulted in a high percentage of differentiation and proliferation of stem cells combined with increased new bone formation. The photothermal properties of the scaffold were analyzed, and under an NIR laser effect (808 nm, 4.6 W/cm^2^) applied for 6 min, a temperature of about 43 °C was obtained. It was concluded that photothermal therapy was successfully used and that it induced cell apoptosis and ablations, resulting in a volume reduction in the cancer tumor. Necrosis of the oncological tissues was also put in evidence. As a general conclusion, the authors stated that these types of implants are suitable for BTE and cancer therapy (Figure 13). Cojocaru et al. [117] conducted *in vivo* tests using [Cs/Col/HyA]/[Fe_3_O_4_] scaffolds with an area of 1 cm^2^ on 40 male Wistar rats with a mean body weight of 165 g to investigate the inflammatory effect of the magnetic implant. All animal procedures were conducted in accordance with ISO 10993-2 [223]. The surgery generated a 1 mm diameter pocket between the hypodermis and dermis, where the scaffold was inserted. A foreign body reaction was observed on day 2 after the implantation of the samples. The presence of leucocytes, collagen fibers, and fibroblasts characterized the inflammatory process. On day 12, fibroblast development was accompanied by angiogenesis, and on day 64, rare leukocytes, resorption, and integration of the scaffold were put in evidence and led to the appearance of new blood vessels. Kim et al. [93] made magnetic [PCL]/[Fe_3_O_4_] composite scaffolds and implanted four small samples on the lateral back side of the spine of three ten-week-old SD rats with an average weight of 300 g. The animals were sacrificed after 14 days. All the harvested biological samples showed mild or moderate fibrosis and angioblastic differentiation. The MNPs were considered safe because no foreign body reaction occurred.

**Figure 14:**
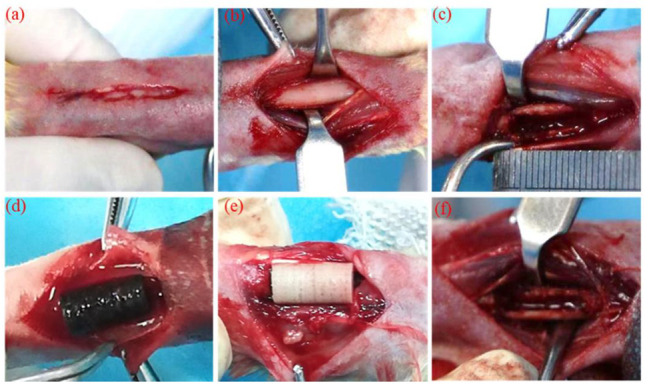
Surgical intervention for scaffold insertion into a bone defect and its steps: (**a**) skin incision; (**b**) rabbit radial diaphysis; (**c**) setting of the bone defect; (**d**) scaffold insertion; (**e**) bone defect with scaffold; (**f**) bone defect without scaffold [96]. Reprinted from [96], Copyright (2023), with permission from Elsevier.

Shuai et al. [96] implanted magnetic [PLLA/PGA]/[Fe_3_O_4_] scaffolds into rabbit radius bone defects (Figure 14). They selected 18 NZW rabbits, and after the surgery, the animals were sacrificed at 1 or 2 months post intervention. No infections were reported, and in the case of implants with 7.5% wt. MNPs, an important quantity of new bone linked to the host bone combined with new blood vessel apparition was noticed. It was concluded that the incorporation of MNPs is strongly related to cell proliferation and adhesion and that the developed scaffolds are an ideal candidate for BTE. Singh et al. [140] developed [PCL]/[MNPs] composite scaffolds with a volume of 1.5 cm × 1.5 cm × 300 μm and surgically inserted them in pouches placed near the spine of SD rats. After 4 weeks, the animals were euthanized. Connective tissues formed along the collagen fibers and localized between the scaffold and neighborhood host tissue, and neovascularization was put in evidence. The scaffolds with the highest percentage of MNPs showed traces of degradation, with the missing areas replaced by fibroblasts. It was concluded that the MNPs promoted angiogenesis because signs of blood vessel formation were present in the histological investigations.

Yun et al. [207] investigated the osteogenic properties of [PCL]/[Fe_3_O_4_] scaffolds under the effect of a static magnetic field. They implanted samples with 10% wt. MNPs and MNP-free samples into 5 mm diameter calvarial defects surgically induced in female 6-to-8-week-old ICR mice. After the procedure, the mice were placed in cages that contained two permanent magnets set in opposition. The generated magnetic field had an average magnetic induction of 15 mT with variable values ranging from 0.05–0.2 mT in the middle of the cage to 15–25 mT in the vicinity of the magnets. After 6 weeks, the animals were euthanized. Microcomputed tomographic and histological analyses showed that the combined effect of the external and internal magnetic fields stimulated new bone formation and proved an adequate strategy in regenerative medicine for bone. Meng et al. [224] prepared [PLA/nHAp]/[γ-Fe_2_O_3_] composite nanofibrous scaffolds using electrospinning. Scaffolds were introduced in lumbar transverse defects in 24 NZW rabbits. Their cages were equipped with permanent magnets to create static magnetic field stimulation. Micro-CT measurements on samples harvested on day 110 after the implantation surgery put in evidence well-organized and homogenous new bone tissue. It was noticed that the external magnetic field accelerated bone remodeling and new bone development. The *in vivo* biocompatibility was evaluated through measurements of biochemical parameters such as creatinine kinase (CK), creatinine (CR), alkaline phosphatase (ALP), and alanine aminotransferase (ALT). All the markers had normal values, showing that the developed scaffolds did not exhibit harmful effects on the surrounding tissues and animal organs. Russo et al. [68] created a defect in rabbit femoral condyle and investigated bone regeneration through magnetic activation. They developed [Col/HAp]/[7% wt. MNPs] based on freeze drying and infiltration methods, which were implanted *in vivo*, together with a cylindrical sample of NdFeB characterized by a height of 8 mm, a diameter of 2 mm, and a magnetic flux density of 1.2 T. A permanent magnet was introduced in a titanium capsule with a thickness of 200 μm to increase the biocompatibility of the device. After careful biomechanical, histological, and histomorphometric investigations conducted 12 weeks after surgery, a pellicular bone structure with interconnected trabeculae oriented perpendicular to the magnetic field lines was put in evidence. This effect occurred because fibrin and collagen can be orthogonally oriented under the influence of an applied magnetic field. This investigation proved that the applied scaffold is an efficient tool for accelerating bone healing.

All the *in vivo* studies mentioned above present necessary steps in BTE and underline the effect of the intrinsic magnetic field of MNPs or an external static magnetic field in bone remodeling and new bone formation. All the presented strategies have been proven very efficient and showed high *in vivo* compatibility.

## 8. Potential Clinical Applications of Polymeric Scaffolds Loaded with MNPs

The main potential clinical applications of magnetic polymeric scaffolds is in the bone tissue engineering domain. Increased adhesion, differentiation, and growth of osteoblasts and fibroblasts were obtained when they were subjected to the effect of a magnetic field, which can be due only to the presence of MNPs or the effect of an external EMF. As mentioned before, tissue engineering is a real challenge because it is still very difficult to manufacture large and complex functional tissues such as bone, heart, kidney, parts of the bloodstream, and muscles [225]. The prevascularization of scaffolds represents an important challenge, and preliminary *in vitro* and *in vivo* studies are necessary to investigate cell growth at a required density and metabolic activity [226,227]. Another challenge is loading of implanted scaffolds with different biological agents [228,229,230,231]. Furthermore, the addition of MNPs to scaffolds can attract cells or growth factors that can be linked to MNPs. Elfick et al. [232] investigated the role of stem cells used in the tissue repair process. They put in evidence a transgenic approach suitable for MSC magnetization. This cell line was modified based on a transfection procedure with the *mms6* gene, with *Magnetospirillum magneticum* AMB-1 as its origin. After this process, the intracytoplasmic MNPs were bioassimilated into modified MSCs. In this way, cellular processes such as proliferation, differentiation, and migration become visible through magnetic resonance devices. Soon, biological MNPs can be included in the scaffold because they represent a non-toxic alternative to the classical FDA-approved iron oxide MNPs.

Russo et al. [233] presented an interesting approach regarding the internal fixation of magnetic scaffolds using magnetic forces. Different configurations were proposed and analyzed based on finite-element modeling. The system comprised a magnetic ring positioned around the leg, four small magnetic pins implanted under the scaffold in the bone, and four stainless-steel pins introduced in the same fashion and magnetized under the influence of an external field; the authors concluded that this system is very efficient, with a beneficial effect in large defect regeneration. This direction must be considered in the near future for magnetic scaffold fixation.

Porous scaffolds are among the most important templates for cell growth and tissue development. Tampieri et al. [234] developed porous bio-hybrid scaffolds made from HAp/Col in which they directly attached MNPs based on impregnation with the ferrofluid method during HAp nucleation. This approach increased the implant biocompatibility because MNPs became an internal component of the scaffold. Magnetically guided tissue development can be foreseen from the mentioned study. Although many synthetic or natural polymers have been used in scaffold manufacturing, challenges such as limited cell density and active cell growth control must be underlined. As mentioned in Section 6 and Section 7, magnetic scaffolds can be sensitive to mechanical stimulation due the interaction between MNPs and magnetic fields. Furthermore, the application of an EMF can increase the osteogenesis and angiogenesis processes by a significant amount.

Other important applications of magnetic polymeric scaffolds are drug delivery and enzyme immobilization. A pulsatile release of drugs that mimics the profile of a given hormone or peptide can be linked to the ideal zero-order release of the active substance over a long time [235]. De Paoli et al. [236] investigated the release of dextran from magnetic [Col/MNPs] scaffolds. They concluded that applying a low-frequency alternative magnetic field enhanced the drug effect. Thermosensitive scaffolds were reinforced with MNPs to achieve controlled drug release due to the thermal properties of the particles that can change the implant temperature. Two directions are reported in the literature with respect to dendrimers and hydrogel solutions [237]. In both cases, a molecular collapse around an average temperature of 42 °C was reported. It was noticed that under the influence of a high-frequency magnetic field, a potential remote release of chemical factors stimulative for tissue regeneration from MNP-loaded drugs and scaffold matrix occurs. Meikle et al. [238] analyzed the functionalization of MNPs with thermoresponsive poly(epsilon-lysine) dendrons tethered with carboxy betaine. They succeeded in delivering vascular endothelial growth factors important in angiogenesis. This process was directly linked to the mild magnetic hyperthermia pulses of MNPs obtained due to an external alternating magnetic field. It was shown in [239] that magnetic alginate scaffolds that suffered a large deformation and a volume increase of 70% due to a medium-frequency magnetic field provided a controlled release of mitoxantrone and chemokine. Magnetic scaffolds can be used to immobilize and release different enzymes that are covalently bonded with a polymeric matrix or MNPs. Under the effect of an alternating magnetic field that generates an imposed temperature value, the enzymes are released. Magnetic scaffolds can exhibit multiple interactions with immobilized enzymes that can improve their thermal stability and restrict the modifications suffered by the molecules during the heating process [240]. Magnetic polymeric scaffolds can be populated with natural or gene-engineered stem cells or signaling molecules. The most used types of cells are multipotent, pluripotent, and progenitor stem cells [241,242,243,244]. Unfortunately, this therapy is incipient for safety reasons because some studies showed contradictory effects regarding anti- and protumor development [245].

Another critical potential clinical application of magnetic polymeric scaffolds is bone tumor treatment. This process is challenging due to a vicious cycle between new bone formation and tumor cell proliferation [246]. Regarding chondrosarcoma, osteosarcoma, and chordoma, which are the most frequently encountered oncological diseases, a release of osteoblast transmembrane molecule (RANKL) directly linked to osteoclast differentiation and activation of the osteolysis process determines healthy bone destruction combined with cancer cell proliferation [83,247,248,249,250]. As mentioned in Section 4, hyperthermia is a direct application that can be used alone or in combination with chemotherapy to increase oncological tissue sensitivity to chemotherapeutic drugs [251,252]. When MNPs were mixed with HAp, the tumor dimensions were drastically reduced due to locally induced heat generation. This effect was amplified by an external magnetic field or a laser light [253]. Matsumine et al. [254] proved that hyperthermia is similar to radiotherapy in treating bone tumor apparition after surgery. Many *in vitro* and *in vivo* studies have demonstrated the efficiency of hyperthermia treatments in cancer therapy. The main challenge associated with this method is exploiting its advantages to adjust the Curie temperature of MNPs to a level superior to the hyperthermia temperature [255,256,257,258] in order to avoid the transition of the MNPs’ magnetization state from superparamagnetic or ferromagnetic to a paramagnetic state.

**Figure 15:**
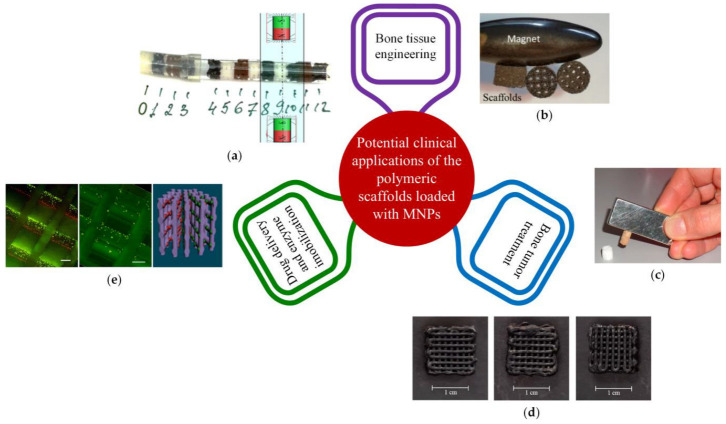
Applications of magnetic scaffolds: (**a**) 3D-printed [PCL/HAp]/[SPIONs] scaffold for BTE [66]; (**b**) SLS-prepared [PLLA]/[Fe_3_O_4_] scaffolds for enhanced cellular activity in BTE [192]; (**c**) freeze-drying manufactured [Col]/[FeHAp] scaffold for cell proliferation and regenerative process [244]; (**d**) 3D-printed [CS/PVA/HAp]/[SPIONs] scaffold for magnetic hyperthermia [250]; (**e**) 3D-bioprinted [PCL/FeHAp] scaffold for 3D patterning of cells [69] ((**a**,**b**,**d**,**e**) are licensed under CC-BY 4.0. (**c**) is adapted with permission from [244]. Copyright 2023 American Chemical Society).

All the potential clinical applications described in this paper are part of a new research area dedicated to the design of magnetic polymeric scaffolds that can enhance osteogenesis and angiogenesis or be involved in hard tissue regeneration after surgical intervention in the absence of or under the effect of an external electromagnetic field. Previously developed applications of the implants mentioned above are presented in Figure 15.

## 9. Conclusions and Future Perspectives

This review presents a new perspective regarding the incorporation of MNPs in BTE scaffolds, providing implants with increased *in vitro* cell performance, *in vivo* efficacy, and good mechanical properties. We showed that a direct consequence of the interaction between MNPs and cells consists of improved osteogenic and angiogenic differentiation. Many *in vitro* studies put in evidence that endothelial cells and osteoblasts can internalize SPIONs or MNPs, leading to new bone formation and blood vessel apparition. We conclude that innovative cell-based regenerative strategies can be applied when an external EMF is applied. These treatments are suitable for bone cancer therapies, such as photothermal and magnetic hyperthermia effects. *In vivo* analysis proved the efficiency of the abovementioned methods by highlighting the significant reduction in tumors and the formation of new bone in their place. The use of highly biocompatible and biodegradable polymers represents an important advantage because secondary surgeries for scaffold removal become unnecessary, and the chemical compounds of the implant matrix are directly related to increased osteogenesis.

The literature reports that, from a biological point of view, the incorporation of MNPs in polymeric scaffolds leads to implants with superior properties. However, additional research must be conducted to completely elucidate the magnetization and demagnetization processes of the MNP effect on cell proliferation and differentiation. In this review paper, we state that magnetism is a key factor, but no existing study defines the intracellular pathways that are influenced by it in a clear and detailed manner. This could represent a future perspective in magnetic-assisted biological environmental analysis.

We investigated whether the concentration of MNPs is an essential factor related to their well-known toxicity. Our literature review revealed that the magnetic properties of scaffolds, which considerably influence osteointegration or cancer cell-death treatment, must be carefully controlled because, in some cases, *in vitro* studies showed a decrease in cell viability, putting in evidence the existence of ROS species and so-called ferroptosis. Although the latter process is considered beneficial in cancer treatments, it can also induce damage to healthy tissues, as a direct consequence of which the influence of the MNP must be further investigated.

Additional analyses are also necessary regarding magnetic scaffolds populated with stem cells or growth factors because these can be magnetically controlled to differentiate and aid in the restoration and regeneration of large bone defects in specific cell lines when autografts or allografts are not a feasible treatment strategy.

The current study is subject to some limitations, such as the safety limit of MNPs’ toxicity for each biomedical application, owing to the hyperthermia effect activated through the thermal properties of MNPs or SPIONs. We consider that a local and personalized treatment can be applied to address the oncological problem or to assist in the patient’s osteogenesis and angiogenesis. Additionally, the lack of *in vivo* studies in the literature means that there is a dearth of information regarding the angiogenic properties of magnetic scaffolds. Exploring the effect of magnetic scaffolds on endothelial cell differentiation and proliferation represents a crucial future perspective that must be taken into account.

Potential clinical applications of magnetic polymeric scaffolds that can be combined with magnetically labeled stem cell therapies and small and portable devices, which generate a static or an alternative EMF to enhance the treatment effect, must be further investigated.

## Figures and Tables

**Table 1:** Studies regarding magnetic scaffolds based on biopolymers and MNPs.

Biopolymer Type	Main Scaffold Material	MNP	Material Characterization Methods	*In Vitro* Tests (Cell Lines)	*In Vivo* Tests (Animal Model)	Reference
Natural	Chitosan (CS)/collagen (Col)/nano-hydroxyapatite (nHAp)	Fe_3_O_4_	Microstructural, magnetic, mechanical, porosity, *in vitro* degradation, biomineralization, the release of Ca ions, measurement of swelling ratio, biocompatibility	MC3T3-E1 (rat skull osteoblasts)	Sprague–Dawley (SD) rats/middle ridge of the skull	[85]
	Modified mesoporous bioglass (MBG)/chitosan (CS)	SrFe_12_O_19_	Morphology, microstructural, magnetic, biocompatibility	hBMSCs (human bone marrow mesemchymal stem cells), MG-63 (human osteosarcoma)	SD rats/bilateral critical size calvarial defect	[86]
	CS, hyaluronic acid (HyA), bovine serum albumin (BSA), and gelatin (G)	Fe_3_O_4_	Morphology, microstructural, magnetic, fluid retention, biocompatibility	MC3T3-E1, NHDF (normal human dermal fibroblast)	-	[87]
	Gelatin–siloxane (GS)	Fe_3_O_4_	Microstructural, magnetic, water uptake and scaffold degradation, mechanical, *in vitro* apatite-forming ability	MSCs	-	[88]
	Silk fibroin (SF) protein	Fe_3_O_4_	Magnetic, microstructural, magnetic hyperthermia, thermogravimetric analysis, differential scanning calorimetry, fluorescence microscopy, biocompatibility	MC3T3-E1	-	[89]
Synthetic	Poly(caprolactone) (PCL)	Multifunctional hydroxyapatite nanoparticles dopped with Eu^3+^ and Gd^3+^ (MF-nHAp)	Microstructural, contact angle measurement, mechanical, mineralization	hMSCs (human mesemchymal stem cells)	-	[90]
	PCL	γ-Fe_2_O_3_	Microstructural, morphology, magnetic, biocompatibility	MSCs (ilium bone marrow of miniature pig)	-	[91]
	PCL	FeHAp	Microstructural, mechanical, biocompatibility	BMSCs	Oryctolagus cuniculus rabbit/distal femoral epiphysis	[92]
	PCL	Fe_3_O_4_	Morphological, microstructural, mechanical, magnetic, biocompatibility	MC3T3-E1	SD rats/lateral direction from the spine (subcutaneous)	[93]
	Poly(lactic-*co*-glycolic acid) (PLGA)/PCL	γ-Fe_2_O_3_	Morphological, mechanical, surface, biocompatibility	OriCell SD rat ADSCs (adipose-derived stem cells)	-	[94]
	PCL/mesoporous bioactive glass (MBG)	Fe_3_O_4_	Microstructural, mechanical, ion dissolution and apatite formation, drug loading and release, magnetic hyperthermia, biocompatibility	hBMSCs	-	[95]
	Poly(L-lactic acid) (PLLA)/poly glycolic acid (PGA)	Fe_3_O_4_	Microstructural, mechanical, magnetic, morphological, thermal, biocompatibility	MG63	New Zealand white (NZW) rabbits/right radial diaphysis	[96]
	PLGA	Fe_3_O_4_	Microstructural	-	Specific pathogen-free (SPF) SD rats/anterior maxilla	[97]

**Table 2:** Main polymeric scaffold manufacturing techniques.

Method Type	Method	Advantages	Disadvantages	References
Conventional	Freeze drying	Scaffolds with porous structure, low stiffness, and small pores; elimination of solvent	High energy consumption, cytotoxic solvent use, and long duration of the procedure	[100,101,102]
	Electrospinning	Versatile and low-cost method; scaffolds with fibrous polymeric structure, high surface area, and high porosity	Use of organic solvent; low thickness structures	[103]
	Gas foaming	Scaffolds with sponge-like structure; avoidance of toxic solvent use	Heat developed during the compression molding process, isolated pores, and a continuous skin layer	[104]
	Solvent-casting particulate leaching	High porosity and a controllable pore diameter through salt particle size	Presence of residual solvent; simple geometric structure; low mechanical integrity	[105,106]
	Thermally induced phase separation	Scaffolds with a highly porous nanoscale structure; low-cost method	Use of solvent; small-scale manufacturing	[107]
Advanced	Selective laser sintering Selective laser melting	Support structure is not required; solvent-free method; control of shape architecture and porosity	Difficulty in removing support powder; expensive equipment; high temperature	[109,110,111]
	Stereolithography	Fast method with high resolution; good surface finish	Support structure is required; use of toxic resins; brittleness and low mechanical strength of the scaffold; expensive equipment	[108]
	Fused deposition modeling	Controlled porosity of the structure; solvent-free method; good mechanical properties; low-cost method	Limited choice of filament material; high heat requirements; medium accuracy	[113,114,115]
	Binder jetting	Manufacture of scaffolds with adapted geometry; multilayered structures	Unbounded powder removal; limited pore size configuration; possibility of the binder being dissolved	[2]

**Table 3:** Concentration, particle diameter, and magnetization values of MNPs incorporated in polymeric or composite matrices.

MNP	MNP Concentration	Particle Diameter (nm)	Matrix Material	Magnetization Value [emu/g]	References
Fe_3_O_4_	5%	11	[PCL]	1.6	[93]
	10%			3.1	
Fe_3_O_4_	5%	12	[PCL]	1	[140]
	10%			2.5	
	15%			6.5	
	20%			12	
γ-Fe_2_O_3_	16.4%	8	[PCL/PLGA]	3.56	[94]
Fe_3_O_4_	5%	15–20	[PCL/MBG]	3.1	[95]
	10%			6.2	
	15%			9.3	
Fe_3_O_4_	2.5%	20	[PLLA/PGA]	1.66	[96]
	5%			3	
	7.5%			6	
	10%			8.5	
Fe_3_O_4_	-	-	[CS/Col]	0.025	[85]
SrFe_12_O_19_	1:7 (ratio of MNPs to BG)	Plate-like with 30 nm thickness	[BG/CS]	4.44	[86]
	1:3 (ratio of MNPs to BG)			7.68	

**Table 4:** Mechanical properties of natural and synthetic polymeric scaffolds loaded with MNPs.

Material (Polymeric Matrix/MNPs)	Synthesis Method	Mechanical Test	Mechanical Properties	Impact on Biological Environment	Reference
[CS/Col/nHAp]/[Fe_3_O_4_]	In situ crystallization and freeze drying	Compressive stress (speed: 1 mm/min) at 20% deformation	Compressive strength of 0.465 MPa; compressive modulus of 2.5 MPa	Scaffold reinforced with MNPs exhibited increased mechanical stability that sustained cell differentiation, proliferation, and maturation	[85]
[CS/Col/HyA]/[[Fe_3_O_4_ SPIONs]	Biomimetic coprecipitation process and freeze drying	Primary axial compression test (speed: 1 mm/s) at 20% deformation followed by a supplementary axial compression test (speed: 1 mm/min)	Young’s modulus ranging from 75 Pa to 275 Pa	The scaffold morphology and especially the pore size and dimension correlated with adequate mechanical properties created a favorable medium for cell division	[117]
[GS]/[Fe_3_O_4_]	Sol–gel method combined with freeze drying	Static compression test and dynamic analysis; mechanical spectrometry in a frequency range of 0.1–10 Hz, a force range of 0.001–0.2 N, and maximum allowed strength of 10%	Storage modulus (E’) increased directly proportionally to the MNP content and varied between 100 kPa (0% wt. MNPs) and 450 kPa (3% wt. MNPs); loss modulus (E”) varied between 60 kPa for 0% wt. MNPs and 150 kPa for 3% wt. MNPs	Adding MNPs improved the resistance to deformation against compressive load and elastic behavior; these properties are beneficial for hard tissue development	[88]
[PCL]/[Gd/multifunctional-nHAp]	Electrospinning	Uniaxial failure test at an extension rate of 10 mm/min	Tensile strength of 3.35 MPa	Cell proliferation and protein absorption improved when MNPs were added	[90]
[PCL/FeHAp]	Rapid prototyping	Compression test performed at a speed of 1 mm/min and with a strain limit of 50%; indirect tensile test	Stress value at a displacement of 0.1 mm of 2 MPa	The implants exhibited high potential for tissue regeneration	[92]
[PCL]/[[Fe_3_O_4_]	Coprecipitation process and freeze drying	Static and dynamic mechanical analysis; mechanical spectrometry in a frequency range of 0.5–10 Hz for 10 min at room temperature	Elastic modulus of 1.4 MPa (5% wt. MNPs) and of 2.4 MPa (10% wt. MNPs)	The scaffolds proved an intense osteogenic differentiation process	[93]
[PLGA/PCL]/[γ-Fe_2_O_3_ SPIONs]	Electrospinning and layer-by-layer assembly of nanoparticles, followed by freeze drying	Atomic force microscopy and force spectroscopy	Young’s modulus of 1.3 GPa	The presence of nanoparticles was beneficial for cell adhesion due to the increase in surface roughness	[94]
[MBG/PCL]/[Fe_3_O_4_]	3D Printing	Static compressive strength test at a speed of 0.5 mm/min and 5 kN	Compressive strength increased proportionally to the MNPs content and varied from 13.9 MPa (5% wt. MNPs) to 16.6 MPa (15% wt. MNPs)	The inclusion of MNPs stimulated cell proliferation and differentiation	[95]
[PLLA/PGA]/[Fe_3_O_4_]	Selective laser sintering	Compressive strength test at a speed of 0.5 mm/min	Compressive strength and Young’s modulus increased proportionally to the MNPs content and varied from 22.6 MPa/2 GPa (0% wt. MNPs) to 41 MPa/3.57 GPa (7.5% wt. MNPs)	Higher cell proliferation capabilities were put in evidence	[96]
[PCL]/[Fe_3_O_4_]	Electrospinning	Tensile mechanical test at a speed of 10 mm/min	Tensile strength increased with the MNPs content from 11.5 MPa (0% wt. MNPs) to 26.2 MPa (15% wt. MNPs); the addition of 20% MNPs resulted in a decrease in the tensile strength at 9.5 MPa	Good bone–cell proliferation was observed, and it was concluded that the scaffold possessed important properties for bone regeneration	[140]

**Table 5:** Cell viability, cell proliferation, and bone markers of some magnetic polymeric scaffolds used in BTE.

Material (Polymeric Matrix/MNPs)	Cell Type	Cell Viability and Proliferation	Bone Marker Control Values/Magnetic Sample Values					Reference
[PCL-P/G]/[Fe_2_O_3_]	hDPSCs	Higher values of optical density (OD) measurements on days 3, 7, and 12 (1/1.3/1.5) showed increased cell viability and proliferation (OD control values: 0.4/0.5/0.6)	ALP (ng/mg)	RUNX2 (r.u.)	BMP2 (r.u.)	OCN (r.u.)	COL1 (r.u.)	[168]
			7 days 1.5/6.5 21 days 4/15	0.9/4.2	0.4/3.5	0.5/3.9	0.4/5	
[PCL]/[Fe_3_O_4_]	MSCs	Adhesion, spreading, and penetration of MSCs were enumerated at 2, 4, and 8 h; 8 h: 80%/95%	ALP (ng/mg)	OPN (fold)	BSP (fold)		COL1 (fold)	[140]
			7 days 0.08/0.09 14 days 0.1/0.2	7 days 1/0.4 14 days 1.2/1.5	7 days 1/2 14 days 0.8/1.1		7 days 1/1.25 14 days 1.48/1	
[PLLA/PGA]/[Fe_3_O_4_]	MG63	CCK-8 assay on days 1, 4, and 7 (absorbance at 490 nm (au)); 1 day: 100%/100%; 4 days: 100%/145%; 7 days: 100%/135%	ALP (μM/min/mg)					[96]
			7 days 0.38/0.45 14 days 0.62/0.92					
[MBG/PCL]/[Fe_3_O_4_]	hBMSCs	CCK-8 assay on days 1, 3, and 7; 1 day: 0.15/0.15; 3 days: 0.28/0.35; 7 days: 0.35/0.7	ALP (μM/min/mg)	RUNX2 (%)	BMP2 (%)	OCN (%)	COL1 (%)	[95]
			7 days 0.38/0.46 14 days 0.55/1.2	7 days 0.1/0.8 14 days 0.25/1.6	7 days 0.1/0.7 14 days 0.25/1.35	7 days 0.4/1.5 14 days 1/3.2	7 days 0.25/1.2 14 days 0.45/2.25	
[PCL]/[MNPs]	MSCs	PicoGreen assay on days 1, 7, and 21: metabolic activity (absorbance at 490 nm (au)); 1 day: 0.5/0.5; 7 days: 0.8/1.1; 21 days: 1.4/1.7	ALP (a.u.)					[91]
			7 days 0.2/0.3 21 days 0.45/0.75					
[MBG/CS]/[SrFe_12_O_19_]	hBMSCs	CCK-8 assay on days 1, 3, and 7 (absorbance at 450 nm (au)); 1 day: 0.4/0.51; 3 days: 0.6/0.75; 7 days: 0.65/1.1	ALP (r.u.)	RUNX2 (r.u.)	OCN (r.u.)		COL1 (r.u.)	[86]
			14 days 1/1.35	14 days 1/1.4	14 days 1/1.5		14 days 0.9/1.4	

**Table 6:** Magnetic polymeric scaffolds under EMF exposure in BTE.

Material (Polymeric Matrix/MNPs)	Synthesis Method	EMF Characteristics	Cell Types	Biological Response	Reference
[PCL]/[Co_0.6_Zn_0.4_Fe_2_O_4_]	Electrospinning	Helmholtz coil system (12.7 cm diameter circular coils); magnetic flux density of 0.1 mT; frequency of 15 Hz; exposure time of 7 h/day; total exposure time of 14 days	L929 (mouse fibroblast cells)	In the absence of an EMF, no important differences in the viability of cells cultured on fibrous scaffolds with 1% wt., 3% wt., and 6% wt. MNPs were noticed. Asignificant improvement in cell metabolic activity was observed in all cases when an EMF was applied. Co_0.6_Zn_0.4_Fe_2_O_4_ exhibited important biocompatibility properties and was proven to stimulate cell proliferation and adhesion.	[203]
[Alginate]/[Fe_3_O_4_]	Freeze drying	Helmholtz coil setup; frequency of 40 Hz; sinusoidal waveform; magnetic induction of 10–15 Gs; total exposure time of 7 days	Ecs (bovine aortic endothelial cells)	Cell metabolic activity was improved between day 3 and day 7 of EMF stimulation. After this time, under the no-EMF condition, it decreased to its initial value by day 14. It was concluded that EMF stimulation combined with MNPs has a positive effect on cell activity.	[204]
[SF]/[CoFe_2_O_4_]	Electrospinning	Permanent magnets with a maximum magnetic field strength of 230 Oe, frequency of 0.3 Hz	MC3T3-E1	Dynamic EMF stimulation improved cell viability, as well as cell proliferation rate and differentiation properties. The cell metabolic activity was sustained by the magnetostriction of MNPs and by the piezoelectricity exhibited by the SF.	[205]
[Fe-doped HAp/PCL]/[commercial Chemicell fluorescent MNPs]	Injection, extrusion, and deposition of fibers combined with the 3D bioprinting technique	NdFeB permanent magnet with 1.2 T magnetic remanence placed under the prepared culture dish	MSCs, human umbilical vein endothelial cells (HUVECs)	The viability and proliferation of cells were good in the case of magnetic scaffolds, and tissue-type tubular-like structures were noticed on fibrous scaffold surfaces covered with HUVECs, proving that the environment was adequate for osteogenesis and angiogenesis. The authors showed that the developed implant permitted magnetic manipulation of the vasculogenic and osteogenic cells.	[69]
[SF/CS]/[Fe_3_O_4_]	Freeze casting	A constant magnetic field with a magnetic flux density of 3 mT	MG63	The scaffold exhibited good biocompatibility, and the application of a low magnetic field showed had beneficial influence on cell proliferation.	[206]
[PCL]/[Fe_3_O_4_]	Freeze drying	NdFeB disc magnet (1 mm thickness × 15 mm diameter) were placed below the culture plates to expose the cell to a north magnetic field; magnetic flux density of 15 mT	Primary mouse calvarium osteoblasts from Institute of Cancer Research (ICR) mice; HUVECs	A static magnetic field enhanced osteoblastic differentiation. Activation of integrin signaling pathways, phosphorylation of Smad 1/5/8, and upregulation of BMP2 were observed. Regarding the proliferation and differentiation of HUVECs under the influence of the magnetic field, an adequate angiogenic response was observed.	[207]
[nHAp/PLLA]/[Fe_2_O_3_]	Low-temperature rapid prototyping	Pulse EMF with magnetic induction of 100 mT obtained from a CLM-B-type pulse magnetic field therapy	Rabbit BMSCs	Under the influence of a pulse EMF, osteogenic differentiation was improved. Fe_2_O_3_ nanoparticles bind to the cell surface regulated and controlled the cell activity under the EMF effect.	[208]
[PLLA]/[Fe_3_O_4_]	Electrospinning	A static magnetic field with magnetic induction of 100 mT	MC3T3-E1	Due to the magnetic feature of PLLA/Fe_3_O_4_ under a static magnetic field, an enhanced proliferation of osteoblasts was put in evidence.	[209]

## Data Availability

Not applicable.
